# Genome-Wide Associations between Genetic and Epigenetic Variation Influence mRNA Expression and Insulin Secretion in Human Pancreatic Islets

**DOI:** 10.1371/journal.pgen.1004735

**Published:** 2014-11-06

**Authors:** Anders H. Olsson, Petr Volkov, Karl Bacos, Tasnim Dayeh, Elin Hall, Emma A. Nilsson, Claes Ladenvall, Tina Rönn, Charlotte Ling

**Affiliations:** 1 Department of Clinical Sciences, Epigenetics and Diabetes, Lund University Diabetes Centre, Clinical Research Centre, Malmö, Sweden; 2 Department of Clinical Sciences, Diabetes and Endocrinology, Lund University Diabetes Centre, Clinical Research Centre, Malmö, Sweden; Albert Einstein College of Medicine, United States of America

## Abstract

Genetic and epigenetic mechanisms may interact and together affect biological processes and disease development. However, most previous studies have investigated genetic and epigenetic mechanisms independently, and studies examining their interactions throughout the human genome are lacking. To identify genetic loci that interact with the epigenome, we performed the first genome-wide DNA methylation quantitative trait locus (mQTL) analysis in human pancreatic islets. We related 574,553 single nucleotide polymorphisms (SNPs) with genome-wide DNA methylation data of 468,787 CpG sites targeting 99% of RefSeq genes in islets from 89 donors. We identified 67,438 SNP-CpG pairs in *cis*, corresponding to 36,783 SNPs (6.4% of tested SNPs) and 11,735 CpG sites (2.5% of tested CpGs), and 2,562 significant SNP-CpG pairs in *trans*, corresponding to 1,465 SNPs (0.3% of tested SNPs) and 383 CpG sites (0.08% of tested CpGs), showing significant associations after correction for multiple testing. These include reported diabetes loci, e.g. *ADCY5*, *KCNJ11*, *HLA-DQA1, INS, PDX1* and *GRB10*. CpGs of significant *cis*-mQTLs were overrepresented in the gene body and outside of CpG islands. Follow-up analyses further identified mQTLs associated with gene expression and insulin secretion in human islets. Causal inference test (CIT) identified SNP-CpG pairs where DNA methylation in human islets is the potential mediator of the genetic association with gene expression or insulin secretion. Functional analyses further demonstrated that identified candidate genes (*GPX7*, *GSTT1* and *SNX19*) directly affect key biological processes such as proliferation and apoptosis in pancreatic β-cells. Finally, we found direct correlations between DNA methylation of 22,773 (4.9%) CpGs with mRNA expression of 4,876 genes, where 90% of the correlations were negative when CpGs were located in the region surrounding transcription start site. Our study demonstrates for the first time how genome-wide genetic and epigenetic variation interacts to influence gene expression, islet function and potential diabetes risk in humans.

## Author Summary

Inter-individual variation in genetics and epigenetics affects biological processes and disease susceptibility. However, most studies have investigated genetic and epigenetic mechanisms independently and to uncover novel mechanisms affecting disease susceptibility there is a highlighted need to study interactions between these factors on a genome-wide scale. To identify novel loci affecting islet function and potentially diabetes, we performed the first genome-wide methylation quantitative trait locus (mQTL) analysis in human pancreatic islets including DNA methylation of 468,787 CpG sites located throughout the genome. Our results showed that DNA methylation of 11,735 CpGs in 4,504 unique genes is regulated by genetic factors located in *cis* (67,438 SNP-CpG pairs). Furthermore, significant mQTLs cover previously reported diabetes loci including *KCNJ11*, *INS*, *HLA*, *PDX1* and *GRB10*. We also found mQTLs associated with gene expression and insulin secretion in human islets. By performing causality inference tests (CIT), we identified CpGs where DNA methylation potentially mediates the genetic impact on gene expression and insulin secretion. Our functional follow-up experiments further demonstrated that identified mQTLs/genes (*GPX7*, *GSTT1* and *SNX19*) directly affect pancreatic β-cell function. Together, our study provides a detailed map of genome-wide associations between genetic and epigenetic variation, which affect gene expression and insulin secretion in human pancreatic islets.

## Introduction

Most cells in the human body share the same genetic sequence while the epigenetic pattern varies between different cell types and over time. DNA methylation is one of the most studied epigenetic modifications and it is involved in multiple biological processes such as transcriptional control during embryonic development, X-chromosome inactivation, genomic imprinting and regulation of cell specific gene expression [1]. In differentiated mammalian cells, DNA methylation occurs primarily on the 5′ position of cytosine followed by guanine, so called CpG sites [2]. Alterations in DNA methylation may affect phenotypic transmission and may be part of the etiology of human disease [3].

Inheritance of epigenetic traits between generations has been shown in animals [4], [5]. Previous studies in twins further suggest that genetic factors may affect DNA methylation profiles [6], [7]. Moreover, genetic variation has been shown to influence the inter-individual variation in DNA methylation in the human brain, fibroblast and adipose tissue [8]–[14]. While some of these studies used the Infinium HumanMethylation27 BeadChip which covers ∼14,500 genes [8]–[10], others used the HumanMethylation450 BeadChip and limited the analysis to *cis* regulatory effects [12]–[14]. However, studies examining the impact of genetic variation on the genome-wide DNA methylation pattern of most genes and regions, in both *cis* and *trans*, throughout the human genome are still scarce.

Pancreatic islets contribute to the regulation of whole body glucose homeostasis by secreting insulin in response to increased plasma glucose concentrations. Deficient insulin secretion, resulting in chronically elevated blood glucose levels, is a characteristic of diabetes mellitus. Recent genome-wide association studies (GWAS) have identified numerous genetic loci associated with diabetes and its related traits [15]–[30]. However, these variants only explain a small proportion of the estimated heritability for diabetes [31], proposing that there are additional genetic factors left to be discovered. These may include genetic variants interacting with epigenetic mechanisms.

**Figure 1:**
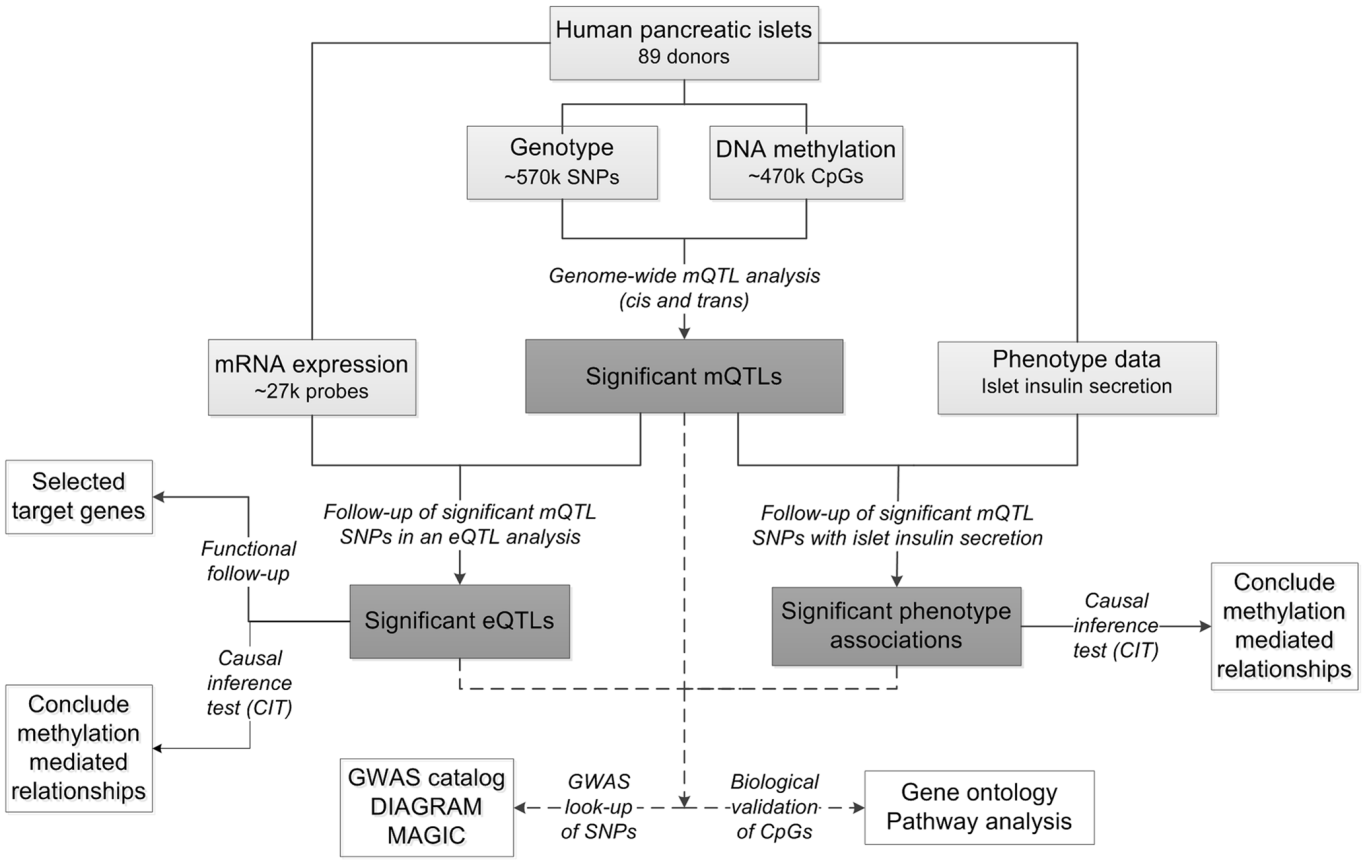
Flow-chart showing the analysis pipeline. Direction of the arrows represents the workflow of the study design with performed analysis indicated. Solid lines indicate analysis performed within data of human pancreatic islets. Dashed lines indicate analysis performed against external databases. Light grey boxes indicate input data of human pancreatic islets. Dark grey boxes indicate output of significant data. White boxes indicate follow-up studies for look-up or functional- and biological validation of significant results.

To study the interaction between genetics and epigenetics and to identify novel loci affecting islet function and potentially diabetes, we performed the first genome-wide DNA methylation quantitative trait locus (mQTL) analysis in human pancreatic islets. The specific goals for this study were to: 1) identify single nucleotide polymorphisms (SNPs) associated with altered DNA methylation (mQTLs) in human pancreatic islets; 2) test if identified SNPs in significant mQTLs affect islet gene expression and diabetes related phenotypes; 3) examine the causal relationship between genotype, DNA methylation and gene expression or insulin secretion in human pancreatic islets; 4) test if identified candidate genes, based on our mQTL results, have a functional role in pancreatic β-cells; 5) examine if mQTLs in human pancreatic islets also associate with diabetes and its related traits in GWAS. To reach these goals, we related genome-wide genotype data of SNPs with genome-wide DNA methylation data of ∼470,000 CpG sites covering 21,231 (99%) RefSeq genes and most genomic regions in pancreatic islets of 89 human donors. Here, both *cis* and *trans* regulatory effects of SNPs on DNA methylation were analyzed. SNPs found to be associated with DNA methylation levels in the mQTL analysis were then followed-up with an expression quantitative trait locus (eQTL) analysis in the human islets, and related to islet insulin secretion data. In addition, we used a causal inference test (CIT) [32] to model the causal relationships between genotype, DNA methylation and phenotypic outcome. A number of candidate genes, where both DNA methylation and gene expression were associated with genetic variation, were then selected for functional follow-up analysis in clonal β-cells. Finally, identified mQTLs were examined for overlap with reported diabetes loci in publicly available GWAS data. The study design is described in **Figure 1**.

Using this approach, we identified significant mQTLs in *cis* and in *trans*. Numerous mQTLs were associated with altered mRNA expression and insulin secretion in human islets. Notably, identified mQTLs covered known diabetes loci. Together, our study highlights the importance of integrating genetic and epigenetic data in order to identify new loci affecting biological processes and disease risk.

## Results

### Associations between genetic variation and DNA methylation – A genome-wide mQTL analysis in human pancreatic islets

To examine whether genetic variation is associated with DNA methylation levels in human pancreatic islets, a genome-wide mQTL analysis was performed. In total, genotype data of 574,553 SNPs and DNA methylation data of 468,787 CpG sites from pancreatic islets of 89 human donors (**Table S1**) were included in the analysis. A correlation heatmap illustrating the overall variability in DNA methylation among included samples is presented in **Figure S1**. In the mQTL analysis, a total of 111,360,152 SNP-CpG pairs were found to be located in *cis* and 269,231,617,059 SNP-CpG pairs were located in *trans*. We proceeded to calculate the statistical significance threshold for the *cis* and *trans*-mQTL analyses, taking the linkage dependency of SNPs and number of tests into account. Linkage disequilibrium (LD) based SNP pruning, which takes into account the linkage dependency of SNPs that are run against DNA methylation of the same CpG site in the mQTL analysis, was then used to calculate the number of independent tests based on r^2^<0.9 for the SNPs and thereby the significance threshold after correction for multiple testing. After LD-based pruning, 102,307,720 SNP-CpG pairs were identified showing independence based on r^2^<0.9 in *cis* and this number was subsequently used as a correction value for multiple testing in the *cis*-mQTL analysis (significance threshold in the *cis*-mQTL: 0.05/102,307,720 = 4.9×10^−10^) (**Table 1**). Furthermore, 200,388,516,440 SNP-CpG pairs were identified showing independence based on r^2^<0.9 in *trans* and this number was used as a correction value for multiple testing in the *trans*-mQTL analysis (significance threshold in the *trans*-mQTL: 0.05/200,388,516,440 = 2.5×10^−13^) (**Table 1**).

**Table 1:** Number of significant mQTL results in human pancreatic islets.

	*cis* -mQTL	*trans* -mQTL
SNP-CpG pairs	67,438	2,562
SNP-CpG pairs with LD r^2^<0.9	31,313	837
SNP-CpG pairs with LD r^2^<0.8	24,963	629
Unique SNPs	36,783	1,465
Unique SNPs with LD r^2^<0.9	20,251	620
Unique SNPs with LD r^2^<0.8	16,557	492
Unique CpG sites	11,735	383
Unique genes	4,504	247

Significance threshold <0.05 after correction for multiple testing.

Correction value *cis* = 102,307,720.

Correction value *trans* = 200,388,516,440.

LD = linkage disequilibrium.

Note that LD-based SNP pruning was used in order to calculate statistical significance thresholds based on number of independent tests. Our goal was to detect and present SNPs that show significant associations with DNA methylation regardless of linkage dependency and we subsequently included all genotyped SNPs in the mQTL analysis. In the *cis*-mQTL analysis, 67,438 SNP-CpG pairs were identified showing significant associations between genotype and DNA methylation levels after correction for multiple testing. These 67,438 SNP-CpG pairs consist of 36,783 unique SNPs (6.4% of tested SNPs) and 11,735 unique CpG sites (2.5% of tested CpG sites) which are annotated to 4,504 unique genes (**Table 1**). Among the significant *cis*-mQTLs, there are 31,313 SNP-CpG pairs with a LD threshold of r^2^<0.9 and 24,963 SNP-CpG pairs with r^2^<0.8 (**Table 1**). These include 20,251 unique SNPs with LD r^2^<0.9 and 16,557 unique SNPs with r^2^<0.8 (**Table 1**).

**Figure 2:**
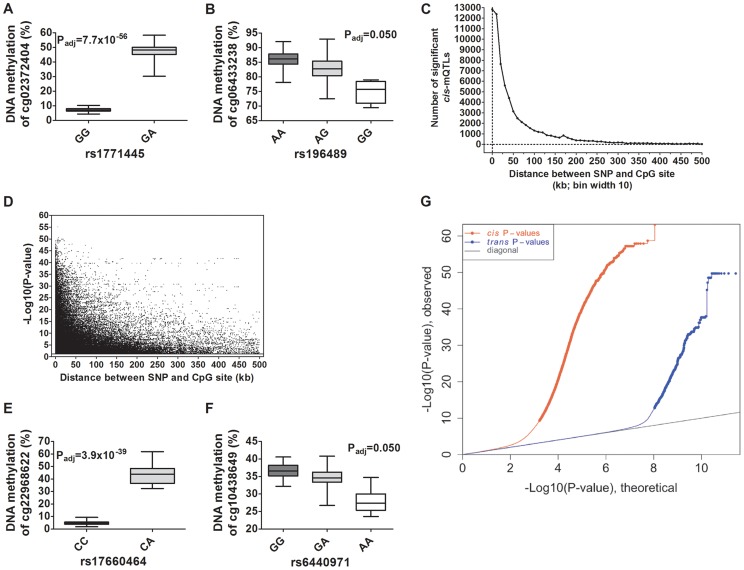
Depiction and distance analysis of associations between genotype and DNA methylation of significant mQTLs in human pancreatic islets. Depiction of (**A**) the most significant *cis*-mQTL; rs1771445 vs. cg02372404, and (**B**) the least significant *cis*-mQTL; rs196489 vs. cg06433283, among all identified *cis*-mQTLs in human pancreatic islets. Data is presented as Box and Whisker plots with P-values adjusted for multiple testing. (**C**) Distance analysis between SNPs and CpG sites of significant *cis*-mQTLs plotted as the number of identified mQTLs within each distance bin. Distance summary: minimum = 0 kb, 10%ile = 1.88 kb, 25%ile = 7.62 kb, 50%ile = 26.31 kb, 75%ile = 74.76 kb, 90%ile = 164.5 kb, maximum = 499.6 kb. (**D**) The strength of associations plotted against the distance between SNPs and CpG sites of significant *cis*-mQTLs after correction for multiple testing. Depiction of (**E**) the most significant *trans*-mQTL; rs17660464 vs. cg22968622, and (**F**) the least significant *trans*-mQTL; rs6440971 vs. cg10438649, among all identified *trans*-mQTLs in human pancreatic islets. Data is presented as Box and Whisker plots with P-values adjusted for multiple testing. (**G**) Quantile-Quantile plots (Q-Q plots) of –log10 (P-values) illustrating the distribution of P-values for all analyzed SNP-CpG pairs in the *cis*- (red dots) and *trans*- (blue dots) mQTL analysis in relation to a theoretical null distribution (grey diagonal line). Bold dots indicate significant mQTLs identified in the *cis*- (red dots) and *trans*-(blue dots) mQTL analysis after correction for multiple testing.

Depictions of the most and least significant *cis*-mQTLs are shown in **Figure 2A–B** and all significant *cis*-mQTLs are presented in **Table S2**. Distance analysis of significant *cis*-mQTLs showed that the majority of associated SNPs were located within a short range from CpG sites (**Figure 2C**). A SNP located within a cytosine or guanine of a CpG site, a so called CpG-SNP, can potentially remove or introduce a CpG site. Among SNP-CpG pairs showing significant associations in the *cis*-mQTL analysis, 459 pairs were identified as CpG-SNPs. Moreover, the *cis*-mQTLs showing the most significant associations were within SNPs located close to a CpG site (**Figure 2D**).

In the *trans*–mQTL analysis, 2,562 SNP-CpG pairs showed significant associations between genotype and DNA methylation levels after correction for multiple testing. These 2,562 SNP-CpG pairs consist of 1,465 unique SNPs (0.3% of tested SNPs) and 383 unique CpG sites (0.08% of tested CpG sites), which are annotated to 247 unique genes. Among the significant *trans*-mQTLs, there are 837 SNP-CpG pairs with a LD threshold of r^2^<0.9 and 629 SNP-CpG pairs with r^2^<0.8 (**Table 1**). These include 620 unique SNPs with LD r^2^<0.9 and 492 unique SNPs with r^2^<0.8 (**Table 1**).

Depictions of the most and least significant *trans*-mQTLs are shown in **Figure 2E–F** and all significant *trans*-mQTLs are presented in **Table S3**. Out of the significant *trans*-mQTLs, 1,564 (61.0%) SNP-CpG pairs, which consist of 970 unique SNPs and 229 unique CpG sites, are located on different chromosomes. Additionally, for the significant *trans*-mQTLs where the SNP and CpG are located on the same chromosome, the median distance between SNP and CpG is 1.2 Mb and these are potentially corresponding to long-range *cis*-effects.

We next generated quantile-quantile (Q-Q) plots of all –log10 (P-values) for the *cis* and *trans* mQTL analyses to illustrate the distribution of the P-values as compared to a theoretical null distribution (**Figure 2G**). The Q-Q plots illustrate that *cis* effects are stronger compared to *trans* effects.

A recent study reports that some probes on Illumina's DNA methylation chip can cross-react to multiple locations in the genome [33]. However, only 14 out of the 11,735 probes used to detect significant *cis*-mQTLs in human islets, and five out of 383 probes used to detect significant *trans*-mQTLs, were demonstrated to have a perfect match elsewhere in the human genome (**Table S2, S3**). Additionally, all significant probes with a 47–50 bp match elsewhere in the genome and possible cross-reactivity based on Chen *et al* [33] have been indicated in **Table S2, S3**.

### Genomic distribution of mQTLs in human pancreatic islets

**Figure 3:**
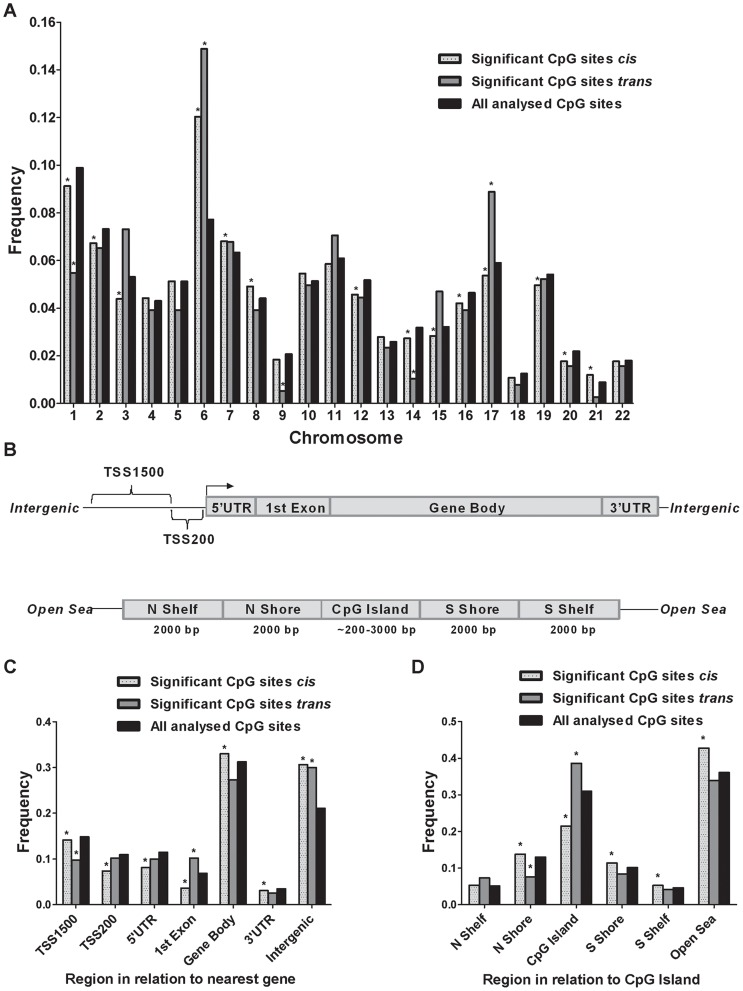
Genomic distribution of CpG sites of significantly identified mQTLs in human pancreatic islets. (**A**) Chromosomal distribution of CpG sites of significant *cis*- and *trans*-mQTLs in comparison to all analyzed CpG sites on the Infinium Human Methylation450 BeadChip. (**B**) All analyzed CpG sites on the Infinium Human Methylation450 BeadChip have been annotated to genomic regions based on their relation to the nearest gene (TSS: proximal promoter, defined as 200 bp or 1500 bp upstream of transcription start site; UTR: untranslated region) or in relation to the nearest CpG island (CpG island: DNA stretch of 200 bp or more with a C+G content of >50% and an observed CpG/expected CpG in excess of 0.6; Shore: the flanking region of CpG islands, 0–2000 bp; Shelf: regions flanking island shores, i.e., covering 2000–4000 bp distant from the CpG island). Distribution of CpG sites of significant mQTLs in relation to (**C**) the nearest gene and (**D**) in relation to CpG islands. *Significantly different distribution (P<0.05) of CpGs of significant *cis*- or *trans*-mQTLs from what is expected by chance based on a Chi-squared-test when compared with all analyzed CpG sites on the Infinium HumanMethylation450 BeadChip.

Although previous cancer studies have described the genomic location of CpG sites that exhibit differential DNA methylation in tumor versus normal cells [34], [35], to our knowledge, no previous study has examined the genomic distribution of CpG sites in genome-wide mQTLs. Moreover, while there is an accumulation of genetic variation on certain chromosomes associated with disease [23], [36], it remains unknown if there is an over- or underrepresentation of significant mQTLs on certain chromosomes linked to islet function. Here, we describe the genomic distribution of significant mQTLs in human pancreatic islets. When analyzing the chromosomal distribution of CpG sites among significant *cis*-mQTLs, an overrepresentation of CpG sites on chromosomes 6, 7, 8 and 21 together with an underrepresentation of CpG sites on chromosomes 1, 2, 3, 12, 14, 15, 16, 17, 19 and 20 were found in comparison to the chromosomal distribution of all analyzed sites on the Infinium HumanMethylation450 BeadChip based on chi-squared-tests (**Figure 3A** **and Table S4A**). In the *trans*-mQTL analysis, an overrepresentation of CpGs was found on chromosomes 6 and 17 together with an underrepresentation on chromosomes 1, 9 and 14 (**Figure 3A** **and Table S4A**). Chromosome 6, which possess the HLA region – a gene region known to be involved in diabetes and autoimmune reaction [37], [38], was found to show the highest enrichment when comparing the chromosomal distribution of CpG sites among significant mQTLs for both the *cis*- and *trans*-analysis compared with all analyzed CpG sites (**Figure 3A** **and Table S4A**).

Moreover, the CpG sites analyzed using the Infinium HumanMethylation 450 BeadChip have been annotated based on their genomic location in relation to the nearest gene (TSS1500, TSS200, 5′UTR, 1^st^ exon, gene body, 3′UTR or intergenic regions) [39] (**Figure 3B**). When comparing the distribution of CpG sites of significant *cis*-mQTLs with all analyzed sites on the Infinium array, CpG sites in the gene body and intergenic regions were found to be overrepresented meanwhile CpG sites in TSS1500, TSS200, 5′UTR, 1^st^ exon and 3′UTR were found to be underrepresented (**Figure 3C** **and Table S4B**). Among significant *trans*-mQTLs, overrepresentations of CpG sites were found in the 1^st^ exon and intergenic regions while an underrepresentation of CpG sites was found in the TSS1500 (**Figure 3C** **and Table S4B**).

The CpG sites analyzed using the Infinium HumanMethylation 450 BeadChip have also been annotated based on their genomic location in relation to CpG islands (CpG island, northern- and southern shores, northern- and southern shelves or open sea) [39] (**Figure 3B**). Overrepresentations of CpG sites were found in northern- and southern shores, southern shelf and open sea while an underrepresentation was found in CpG islands when comparing the location of CpG sites of significant *cis*-mQTLs with all analyzed sites on the Infinium array (**Figure 3D** **and Table S4C**). CpG sites of significant *trans*-mQTLs were found to be overrepresented in CpG islands and underrepresented in northern shores (**Figure 3D** **and Table S4C**).

Epigenetic variation in enhancer regions has been proposed to play a key role in the regulation of gene expression in pancreatic islets [40]–[43]. We therefore proceeded to test if CpG sites in our significant mQTLs are located in long stretch enhancers based on publicly available data for human pancreatic islets [42]. These stretch enhancers are referred to as large gene elements (≥3 kb) of enhancer states that are cell type specific [42]. Here, we found that 993 (8.5%) CpG sites in our significant *cis*-mQTLs and 11 (2.9%) CpG sites in our significant *trans*-mQTLs are located in long stretch enhancers specific for pancreatic islets (**Table S2 and Table S3**), which is not more than expected by chance (P>0.05). Additionally, we found that 139 (1.2%) CpG sites in our significant *cis*-mQTLs and only two CpG sites in the significant *trans*-mQTLs are located in active enhancer regions of pancreatic islets identified by Pasquali et al [43] (**Table S2 and Table S3**).

Moreover, we tested if the genomic distribution of the significant mQTLs found in human islets in our study could be replicated in publicly available data. Here, we took advantage of published mQTL data in adipose tissue from Grundberg *et al* and we analyzed the genomic distribution of their significant *cis*-mQTLs [12]. In agreement with the genomic distribution of significant *cis*-mQTLs in human islets, we found that significant *ci*s-mQTLs in human adipose tissue were overrepresented in the intergenic region, the gene body, the open sea as well as the shore and shelf regions, while underrepresented in regions close to the TSS and CpG island regions (**Figure S2A–B**). On the other hand, we found differences between human islets and adipose tissue regarding the chromosomal distribution of significant *cis*-mQTLs (**Figure 3A** and **Figure S2C**). Of note, differences in the study design and filtering of CpG probes between the two studies may influence these results.

### Association of identified mQTL-SNPs with mRNA expression – A follow-up eQTL analysis in human pancreatic islets

Both genetic variation and DNA methylation have been shown to regulate gene expression [44], [45]. Therefore, SNPs identified to significantly affect DNA methylation in the mQTL analysis were followed-up and related to mRNA expression levels in human pancreatic islets. To calculate the number of independent tests to be used for correction for multiple testing in this analysis, we first connected SNPs of significant *cis*-mQTLs (n = 36,783) with all mRNA transcripts on the Affymetrix array located within 500 kb of respective SNP – the set *cis* boundary distance. With this setting, 895,764 SNP-mRNA transcript combinations were found in *cis*. However, after LD-based pruning of these SNPs, 692,616 SNP-mRNA transcript combinations remained showing independence of SNPs (based on r^2^<0.9) and this number was subsequently used as a correction value for multiple testing (significance threshold in the *cis*-eQTL: 0.05/692,616 = 7.2×10^−8^) (**Table 2**). In this *cis*-eQTL analysis, 302 SNP-mRNA transcript pairs were identified showing significant associations between genotypes and mRNA expression levels after correction for multiple testing (**Table 2** and **Table S5**). These 302 significant pairs consist of 243 unique SNPs (0.7% of the significant *cis*-mQTL SNPs) and 46 unique mRNA transcripts (0.2% of tested mRNA transcripts). Among the significant *cis*-eQTLS, there are 117 SNP-mRNA transcript pairs with a LD threshold of r^2^<0.9 and 86 SNP-mRNA transcript pairs with r^2^<0.8 (**Table 2**). These include 99 unique SNPs with LD r^2^<0.9 and 76 unique SNPs with r^2^<0.8 (**Table 2**).

**Table 2:** Number of significant eQTL results in the human pancreatic islets.

	eQTLs of *cis*-mQTL-SNPs	eQTLs of *trans*-mQTL-SNPs
SNP-mRNA transcript pairs	302	32
SNP-mRNA transcript pairs with LD r^2^<0.9	117	16
SNP-mRNA transcript pairs with LD r^2^<0.8	86	10
Unique SNPs	243	22
Unique SNPs with LD r^2^<0.9	99	10
Unique SNPs with LD r^2^<0.8	76	7
Unique mRNA transcripts	46	8
Unique genes	42	7

Only SNPs of significant mQTLs are included in the eQTL analysis.

SNPs of significant *cis*-mQTLs are regressed against mRNA expression of transcripts located in *cis* (≤500 kb).

SNPs of significant *trans*-mQTLs are regressed against mRNA expression of all transcripts.

Significance threshold <0.05 after correction for multiple testing.

Correction value of eQTL analysis for *cis*-mQTL-SNPs = 692, 616.

Correction value of eQTL analysis for *trans*-mQTL-SNPs = 16,982,420.

LD = linkage disequilibrium.

The SNPs of significant *trans*-mQTLs (n = 1,465) were then related to mRNA expression levels of all transcripts included on the Affymetrix array, giving rise to 40,127,815 SNP-mRNA transcript combinations. The correction value for multiple testing was calculated to 16,982,420 after LD-based pruning of SNPs (based on r^2^<0.9) (significance threshold in the *trans*-eQTL: 0.05/16,982,420 = 2.9×10^−9^) (**Table 2**). In the *trans*-eQTL, 32 SNP-mRNA transcript pairs consisting of 22 unique SNPs (1.5% of the significant *trans*-mQTL SNPs) and 8 unique mRNA transcripts (0.02% of tested mRNA transcripts) were found to show significance (**Table 2** and **Table S6**). Among the significant *trans*-eQTLs, there are 16 SNP-mRNA transcript pairs with a LD threshold of r^2^<0.9 and 10 SNP-mRNA transcript pairs with r^2^<0.8 (**Table 2**). These include 10 unique SNPs with LD r^2^<0.9 and 7 unique SNPs with r^2^<0.8 (**Table 2**).

Moreover, a correlation heatmap illustrating the overall variability in mRNA expression among included samples is presented in **Figure S3**. We next used Mantel's test [46] to compare the hierarchical clustering results for mRNA expression (**Figure S3**) and DNA methylation (**Figure S1**) and obtained a correlation coefficient of 0.21 (P = 0.005).

### Causality inference test (CIT) - DNA methylation potentially mediates the genetic impact on mRNA expression

**Figure 4:**
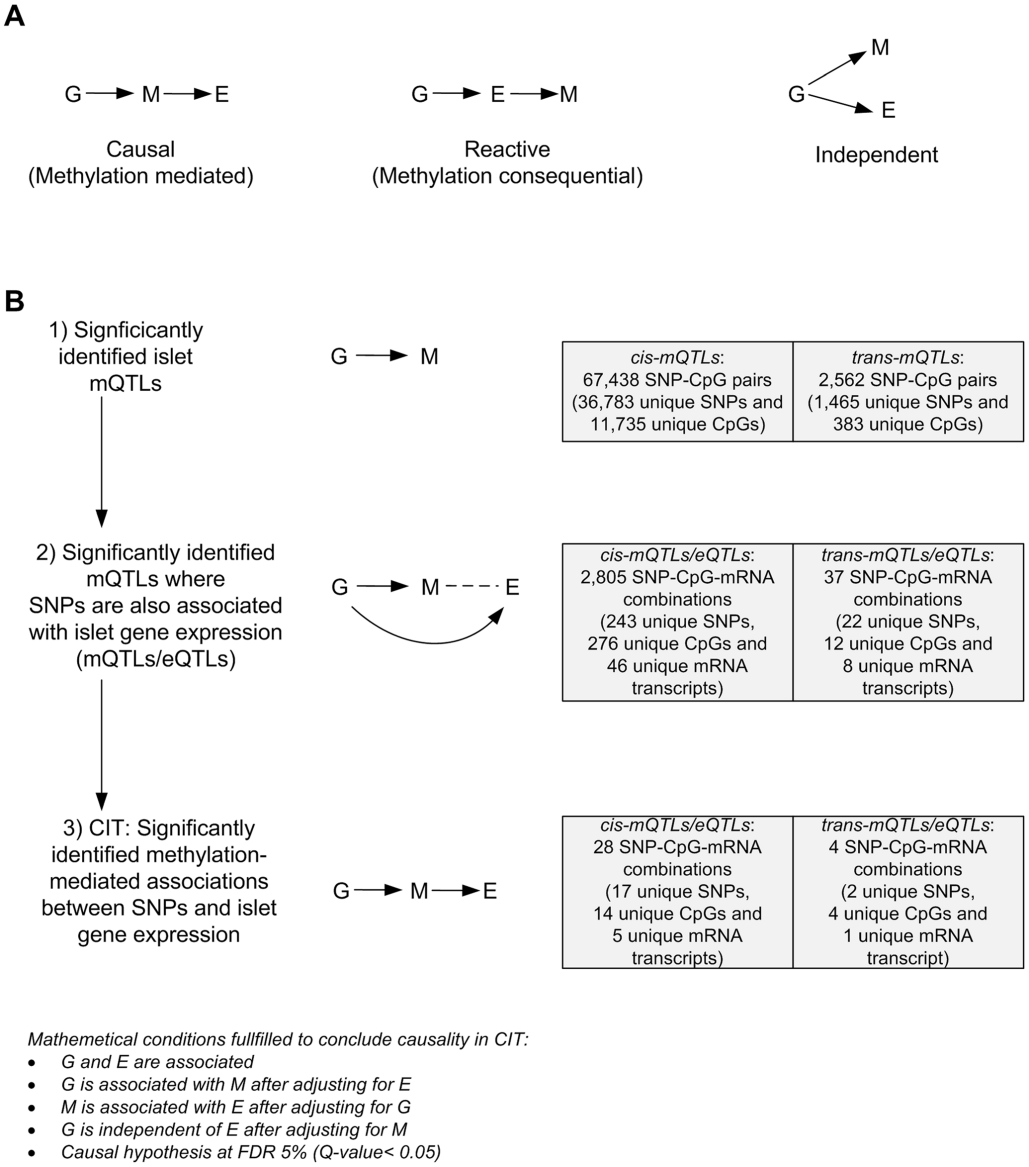
CIT analysis identifies mQTLs where DNA methylation potentially mediates genetic associations with mRNA expression in human pancreatic islets. (**A**) Depiction of possible relationship models between genotype as a causal factor (G), DNA methylation as a potential mediator (M) and islet mRNA expression as a phenotypic outcome (E). Left diagram: The causal or methylation mediated model. Middle diagram: The reactive or methylation-consequential model (reverse causality). Right diagram: The independent model. (**B**) Illustration of the study approach to identify if DNA methylation of CpG sites potentially mediates the causal association between SNPs and islet mRNA expression. Left: Workflow steps. Middle: Tested relationships between G, M and E in the different steps. Right: Number of identified sites in each step. Bottom: Conditions that must be fulfilled to conclude a mathematical definition of a causal relationship between G, M and E. Significantly called as causal at 5% FDR (causal hypothesis Q<0.05).

We further used CIT [32] to examine if relationships between genotypes and phenotype (gene expression) are potentially mediated through DNA methylation of CpG sites in significant mQTLs. In this CIT approach, we consider SNPs identified in the mQTL/eQTL analysis as causal factors (G), DNA methylation of CpG sites identified in the mQTL analysis as potential mediators (M) and mRNA expression identified in the eQTL analysis as a phenotypic outcome (E). The possible relationships between these three factors are shown in **Figure 4A**. Significant SNP-CpG pairs from the mQTL analysis (*Step 1* **Figure 4B**), where the mQTL-SNPs also show significant association with mRNA expression in the eQTL analysis (*Step 2* **Figure 4B**), were included in the CIT. In the CIT analysis of *cis*-mQTLs/eQTLs, we identified 28 SNP-CpG-mRNA combinations (1.0%) consisting of 17 unique SNPs, 14 unique CpG sites and 5 unique mRNA transcripts that were significantly called as causal (causal hypothesis Q-value<0.05 based on FDR) and these represent potential methylation-mediated relationships between SNPs and mRNA expression (*left panel* **Figure 4A**, *step 3* **Figure 4B** and **Table 3**). All hypothesis tests of the CIT for *cis* interactions are presented in **Table S7**. Interestingly, several identified relationships where DNA methylation potentially mediates the causal association between SNP and mRNA expression were annotated to *HLA* genes (**Table 3**), a gene region strongly linked to type 1 diabetes [37]. Moreover, a causal relationship between SNPs, DNA methylation and mRNA expression of genes involved in glutathione metabolism, including *GSTT2* (Q<0.05, **Table 3**) and *GSTT1* (P<0.05, **Table S7**), were also identified in the CIT analysis. Glutathione metabolism is known to protect against oxidative stress [47]–[49] and thereby has a potential role in islet function.

**Table 3:** Identified *cis*-mQTLs/eQTLs where methylation of CpG sites is a potential mediator of genetic association with mRNA expression in human pancreatic islets.

Chr	CpG			SNP	mRNA Transcript		G vs M		G vs E		CIT
	Id	Gene	Gene Region	Id	Id	Gene	T-Stat	Corrected P-Value	T-Stat	Corrected P-Value	Q-Value
6	cg00119778	*-*	Intergenic	rs2395165	8125447	*HLA-DQB1*	10.02	8.00E-08	6.37	7.35E-03	1.61E-03
6	cg00440797	*HLA-DRB5*	Body	rs3130287	8125447	*HLA-DQB1*	9.63	4.56E-07	7.40	7.82E-05	2.94E-03
6	cg25140213	*HLA-DRB6*	Body	rs2395165	8125447	*HLA-DQB1*	9.14	4.27E-06	6.37	7.35E-03	3.28E-03
2	cg01577475	*PAX8;LOC440839*	Body;Body	rs7589901	8044605	*LOC654433*	−9.90	1.37E-07	−6.89	7.66E-04	3.42E-03
22	cg24846343	*DDTL*	Body	rs2330635	8074962	*GSTT2*	−9.91	1.28E-07	7.64	2.63E-05	4.95E-03
6	cg00440797	*HLA-DRB5*	Body	rs3130342	8125447	*HLA-DQB1*	9.63	4.56E-07	7.40	7.82E-05	5.32E-03
6	cg02919082	*HLA-DQA1*	Body	rs3021058	8125447	*HLA-DQB1*	−7.80	1.91E-03	−6.04	3.15E-02	5.93E-03
6	cg02919082	*HLA-DQA1*	Body	rs4642516	8125447	*HLA-DQB1*	−7.80	1.91E-03	−6.04	3.15E-02	5.93E-03
6	cg02919082	*HLA-DQA1*	Body	rs9275141	8125447	*HLA-DQB1*	−7.80	1.91E-03	−6.04	3.15E-02	5.93E-03
6	cg00119778	*-*	Intergenic	rs3129948	8125436	*HLA-DRB5*	7.67	3.36E-03	5.96	4.27E-02	6.14E-03
6	cg02919082	*HLA-DQA1*	Body	rs2856695	8125447	*HLA-DQB1*	−7.80	1.91E-03	−6.04	3.15E-02	6.86E-03
6	cg04461101	*-*	Intergenic	rs3129954	8125436	*HLA-DRB5*	7.97	8.84E-04	5.96	4.27E-02	7.41E-03
6	cg04461101	*-*	Intergenic	rs3129955	8125436	*HLA-DRB5*	7.97	8.84E-04	5.96	4.27E-02	9.54E-03
2	cg12889195	*PAX8;LOC440839;LOC654433*	Body;Body;TSS1500	rs7589901	8044605	*LOC654433*	10.96	1.19E-09	−6.89	7.66E-04	9.96E-03
22	cg24846343	*DDTL*	Body	rs1007888	8071809	*GSTT2*	−11.23	3.56E-10	8.31	1.26E-06	1.61E-02
22	cg24846343	*DDTL*	Body	rs1007888	8074962	*GSTT2*	−11.23	3.56E-10	8.31	1.26E-06	1.68E-02
6	cg04461101	*-*	Intergenic	rs2395165	8125447	*HLA-DQB1*	9.53	7.37E-07	6.37	7.35E-03	1.96E-02
2	cg07594247	*PAX8;LOC440839;LOC654433*	Body;Body;TSS1500	rs7589901	8044605	*LOC654433*	9.17	3.74E-06	−6.89	7.66E-04	2.27E-02
6	cg14645244	*HLA-DRB1*	Body	rs2395165	8125447	*HLA-DQB1*	−8.42	1.16E-04	6.37	7.35E-03	2.77E-02
6	cg00119778	*-*	Intergenic	rs3134954	8125447	*HLA-DQB1*	9.84	1.81E-07	7.40	7.82E-05	3.11E-02
2	cg19083407	*PAX8;LOC440839;LOC654433*	Body;Body;TSS1500	rs4849179	8044605	*LOC654433*	8.79	2.13E-05	−6.93	6.27E-04	3.55E-02
22	cg24846343	*DDTL*	Body	rs4822453	8071809	*GSTT2*	−9.82	1.94E-07	7.28	1.34E-04	3.67E-02
2	cg21482265	*PAX8;LOC440839;LOC654433*	Body;Body;TSS1500	rs4849179	8044605	*LOC654433*	9.24	2.70E-06	−6.93	6.27E-04	3.87E-02
6	cg14645244	*HLA-DRB1*	Body	rs3134603	8125447	*HLA-DQB1*	−10.03	7.42E-08	7.16	2.27E-04	3.94E-02
2	cg17445212	*PAX8;LOC440839;LOC654433*	Body;Body;TSS1500	rs7589901	8044605	*LOC654433*	8.95	1.01E-05	−6.89	7.66E-04	4.02E-02
6	cg11752699	*HLA-DRB6*	Body	rs2395165	8125447	*HLA-DQB1*	9.47	9.60E-07	6.37	7.35E-03	4.17E-02
6	cg00119778	*-*	Intergenic	rs3129955	8125436	*HLA-DRB5*	7.67	3.36E-03	5.96	4.27E-02	4.43E-02
6	cg11752699	*HLA-DRB6*	Body	rs3134954	8125447	*HLA-DQB1*	9.46	1.01E-06	7.40	7.82E-05	4.56E-02

Causal Inference Test (CIT) was used to test if associations between SNPs identified in the *cis*-mQTL analysis and islet mRNA expression was mediated by DNA methylation of CpG sites.

G vs M: Adjusted associations between genotype (G) and DNA methylation (M) – *cis*-mQTL analysis. P-values corrected for multiple testing based on the number of independent tests performed.

G vs E: Adjusted associations between genotype (G) and human islet mRNA expression (E) – eQTL analysis of *cis*-mQTL-SNPs. P-values corrected for multiple testing based on the number of independent tests performed.

CIT: Causal hypothesis at 5% FDR (Q-value<0.05) showing that the relationship between genotype (G) and islet mRNA expression (E) is potentially mediated through DNA methylation (M).

In the CIT analysis of *trans*-mQTLs/eQTLs, we identified 4 SNP-CpG-mRNA combinations (10.8%) showing a causal relationship with FDR<5% (*step 3* **Figure 4B** and **Table S8**).

### Biological features of genes identified in the mQTL/eQTL analyses

Next, we performed gene ontology and KEGG pathway analyses to identify cellular components or biological pathways with enrichment of genes that were significant in the mQTL and/or eQTL analyses in human pancreatic islets.

In the gene ontology analysis of significant *cis*-mQTLs, genes annotated to identified CpG sites were enriched in biological processes of relevance to human pancreatic islets, including the MHC protein complex (P_adj_ = 5.8×10^−7^) and the endoplasmic reticulum (ER) to golgi transport (P_adj_ = 1.6×10^−2^) (**Figure S4**, *includes all enriched biological processes*). Moreover, in the KEGG pathway analysis, type 1 diabetes (P_adj_ = 3.3×10^−7^), phagosome (P_adj_ = 3.0×10^−4^), cell adhesion molecules (P_adj_ = 5.0×10^−4^), extracellular-receptor matrix (ECM) interaction (P_adj_ = 2.7×10^−3^) and folate biosynthesis (P_adj_ = 0.011) were found among the enriched pathways (**Table 4**, *includes all enriched KEGG pathways*).

**Table 4:** KEGG pathways with enrichment of genes annotated to CpG sites of significant *cis*-mQTLs in human pancreatic islets.

Pathway (total number of genes in pathway)	Observed number of genes	Expected number of genes	Ratio of enrichment	Raw *P*-value	Adjusted *P*-value	Observed genes
Type 1 Diabetes (41)	27	9.16	2.95	3.02×10^−9^	3.32×10^−7^	*HLA-DRA, HLA-DQA2, CD86, HLA-DQA1, HLA-E, HLA-F, HLA-DMB, CD80, IL1A, GZMB, HLA-DPB1, INS, HLA-A, FAS, HLA-DMA, HLA-DPA1, HLA-DRB1, HLA-B, ICA-1, HLA-DQB1, HLA-G, PTPRN2, HLA-C, HLA-DOA, GAD1, HLA-DOB, HLA-DRB5*
Autoimmune thyroid disease (41)	27	9.16	2.95	3.02×10^−9^	3.32×10^−7^	*HLA-DRA, HLA-DQA2, CTLA4, CD86, HLA-DQA1, HLA-E, HLA-F, HLA-DMB, CD80, TPO, GZMB, HLA-DPB1, HLA-A, FAS, HLA-DMA, HLA-DPA1, HLA-DRB1, HLA-B, HLA-DQB1, HLA-G, TG, HLA-C, HLA-DOA, TSHR, HLA-DOB, HLA-DRB5, IFNA4*
Allograft rejection (34)	22	7.59	2.90	1.43×10^−5^	1.05×10^−5^	*HLA-DRA, HLA-DQA2, CD86, HLA-DQA1, HLA-E, HLA-F, HLA-DMB,CD80, GZMB, HLA-DPB1, HLA-A, FAS, HLA-DPA1, HLA-DRB1, HLA-DMA, HLA-B, HLA-DQB1, HLA-G, HLA-C, HLA-DOA, HLA-DRB5, HLA-DOB*
Graft versus host disease (37)	23	8.26	2.78	2.19×10^−7^	1.20×10^−5^	*HLA-DRA, HLA-DQA2, CD86, HLA-DQA1, HLA-E, HLA-F, HLA-DMB, CD80, IL1A, GZMB, HLA-DPB1, HLA-A, FAS, HLA-DPA1, HLA-DRB1, HLA-DMA, HLA-B, HLA-DQB1, HLA-G, HLA-C, HLA-DOA, HLA-DRB5, HLA-DOB*
Viral myocarditis (66)	31	14.74	2.10	8.44×10^−6^	0.0003	*HLA-DRA, HLA-DQA2, CASP3, CD55, MYH13, CD86, HLA-DQA1, HLA-E, HLA-F, CASP9, SGCD, HLA-DMB, CD80, CAV1, LAMA2, HLA-DPB1, HLA-A, MYH11, ITGB2, HLA-DPA1, HLA-DRB1, HLA-DMA, HLA-B, FYN, HLA-DQB1, HLA-G, MYH15, HLA-C, HLA-DOA, HLA-DOB, HLA-DRB5*
Phagosome (143)	55	31.94	1.72	9.28×10^−6^	0.0003	*HLA-DRA, DYNC1I2, TUBB2A, ATP6V1A, HLA-E, HLA-F, TAP1, TUBB6, DYNC1I1, ITGB5, HLA-DPB1, NCF2, HLA-A, HLA-DRB1, MBL2, HLA-DPA1, PLA2R1, HLA-DQB1, ITGB3, ATP6V0A4, ATP6V0A2, TAP2, TUBAL3, HLA-C, DYNC2H1, TUBB8, TUBA3D, COMP, ATP6V0D1, ATP6V0E2, ATP6V1G1, C3, HLA-DQA2, PIK3C3, SEC61B, TUBA1A, THBS2, HLA-DQA1, RAB7A, VAMP3, HLA-DMB, TUBA3E, FCGR3B, COLEC11, TLR6, CD36, ITGB2, HLA-DMA, HLA-B, TLR2, HLA-G, SCARB1, HLA-DOA, HLA-DOB, HLA-DRB5*
Cell adhesion molecules - CAMs (125)	49	27.92	1.75	1.54×10^−5^	0.0005	*HLA-DRA, CDH4, CLDN15, CD86, HLA-E, HLA-F, CD276, SDC2, SELL, CNTNAP2, NRXN1, CLDN14, HLA-DPB1, HLA-A, HLA-DRB1, HLA-DPA1, CDH15, CDH1, HLA-DQB1, CD6, CLDN14, HLA-C, CLDN3, NCAM1, ITGA9, CLDN18, HLA-DQA2, CTLA4, CLDN23, HLA-DQA1, HLA-DMB, ICAM3, CD80, MAG, JAM3, NEGR1, ITGB2, CNTNAP1, PTPRF, HLA-DMA, HLA-B, MPZ, CDH5, HLA-G, NFASC, HLA-DOA, HLA-DOB, SIGLEC1, HLA-DRB5*
Extracellular matrix (ECM) receptor interaction (83)	34	18.54	1.83	0.0001	0.0027	*COL-4A2, AGRN, HSPG2, TNXB, SDC2, ITGB5, COL6A3, ITGA3, GP5, COL6A1, ITGB3, COL5A1, COL2A1, ITGA11, COL4A1, COL5A3, ITGA1, CD44, ITGA9, COMP, LAMA4, SV2C, COL6A2, LAMC1, THBS2, COL11A2, COL1A1, RELN, LAMA2, CD36, LAMB1, VWF, GP6, LAMA5*
Other types of O-glycan biosynthesis (43)	20	9.61	2.08	0.0004	0.0098	*UGT1A1, GXYLT2, UGT1A4, GXYLT1, UGT2B15, UGT1A10, ST6GAL1, GLT25D2, UGT1A7, MGAT5B, POMGNT1, UGT1A5, ST3GAL3, UGT1A3, UGT1A9, UGT2B17, FUT9, UGT1A6, UGT1A8, ST6GAL2*
Folate biosynthesis (11)	8	2.46	3.26	0.0005	0.0110	*ALPPL2, ALPP, GCH1, QDPR, ALPL, PTS, DHFR, GGH*
Antigene processing and presentation (68)	27	15.19	1.78	0.0009	0.0180	*HLA-DRA, HLA-DQA2, HLA-DQA1, HLA-E, HLA-F, HSP90AB1, TAP1, HLA-DMB, HLA-DPB1, HLA-A, HSPA1B, HLA-DPA1, HSPA1L, HLA-DRB1, HLA-DMA, HLA-B, TAPBP, NFYA, HLA-DQB1, HLA-G, TAP2, HSP90AA1, HLA-C, HLA-DOA, HLA-DOB, KLRC2, HLA-DRB5*

P-values have been adjusted for multiple testing using Benjamini-Hochberg.

In the gene ontology analysis of genes showing differential expression between genotype groups in the eQTL analysis of significant *cis*-mQTL-SNPs, we again found enrichment of genes in the MHC protein complex (P_adj_ = 1.6×10^−3^) and in ER to golgi transport (P_adj_ = 1.4×10^−2^). Moreover, genes involved in glutathione peroxidase activity (P_adj_ = 1.1×10^−2^) and glutathione transferase activity (P_adj_ = 1.1×10^−2^) were enriched in the gene ontology analysis of the *cis*-eQTLs (**Figure S5**). In the KEGG pathway analysis of differentially expressed genes in the *cis*-eQTL (**Table S9**), genes involved in the glutathione metabolism pathway which is of relevance to islet function were enriched including the following identified genes: *GSTT1*, *GSTM3* and *GPX7* (P_adj_ = 3.0×10^−4^).

Furthermore, genes annotated to CpG sites of significant *trans*-mQTLs were also found to be enriched in the MHC protein complex (P_adj_ = 1.1×10^−3^) and the ER part (P_adj_ = 3.8×10^−2^) when performing a gene ontology analysis (**Figure S6**). This was also reflected in the KEGG pathway analysis of *trans*-mQTLs (**Table S10**) where type 1 diabetes was found to be an enriched pathway of relevance in human pancreatic islets, including the following genes: *PTPRN2, HLA-DRB1, HLA-B, HLA-C, HSPD1* and *HLA-DRB5* (P_adj_ = 6.0×10^−4^).

In the gene ontology analysis of genes showing differential expression between genotype groups in the eQTL analysis of significant *trans*-mQTL-SNPs, the carboxylic acid metabolic process was found to be enriched (P_adj_ = 8.4×10^−3^) (**Figure S7**). However, no significant enrichment was found in the KEGG pathway analysis including the same dataset.

### Knockdown of *Gpx7*, *Gstt1* and *Snx19* alters β-cell proliferation and cell death signaling

**Figure 5:**
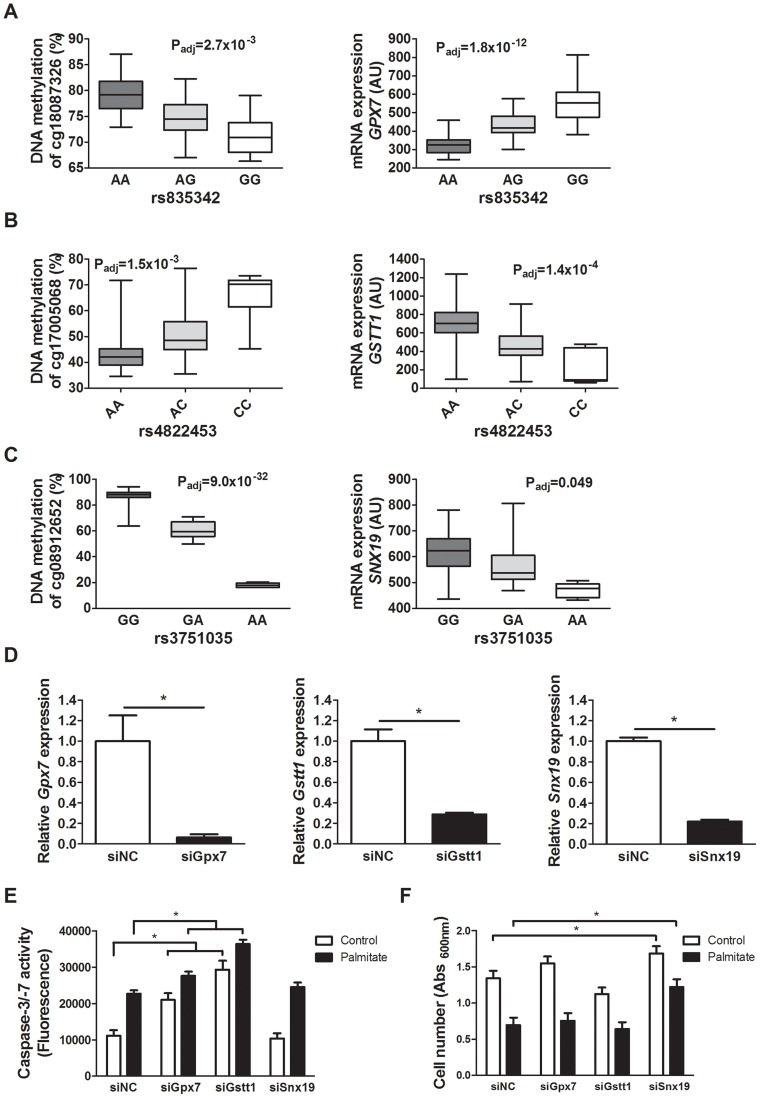
Identified mQTL/eQTL candidate genes *GPX7*, *GSTT1* and *SNX19* affect β-cell number and apoptosis. Associations identified in the mQTL/eQTL analyses of human pancreatic islets. (**A**) rs835342 located approximately 5 kb upstream of *GPX7* associates with DNA methylation of cg18087326 located 406 bp upstream of the *GPX7* transcription start site (TSS) as well as with mRNA expression of *GPX7*. (**B**) rs4822453 located ∼121 kb downstream of *GSTT1* associates with DNA methylation of cg17005068 located 241 bp upstream of the *GSTT1* TSS as well as with mRNA expression of *GSTT1*. (**C**) rs3751035 located within exon 1 of *SNX19* associates with DNA methylation of cg08912652 located within the gene body of *SNX19* as well as with mRNA expression of *SNX19*. Data are presented as Box and Whisker plots with P-values adjusted for multiple testing. (**D**) qPCR quantification of siRNA mediated knockdown of *Gpx7* (siGpx7), *Gstt1* (siGstt1) and *Snx19* (siSnx19) compared to negative control siRNA (siNC). * P<0.01, the graphs show the average of four independent knockdown experiments presented as mean ± SEM. (**E**) Knockdown of *Gpx7* and *Gstt1* resulted in increased combined caspase-3/7 activity compared to negative control siRNA under both control (white bars) and lipotoxic (black bars) conditions. * P<0.05, the graph shows the average of three independent knockdown experiments presented as mean ± SEM. (**F**) Knockdown of *Snx19* (siSnx19) resulted in increased cell number compared to negative control siRNA (siNC) under both control (white bars) and lipotoxic (black bars) conditions. * P<0.05, the graph shows the average of six independent knockdown experiments presented as mean ± SEM.

To examine whether altered expression of some of the identified candidate genes in the islet mQTL/eQTL analyses affect β-cell function and thereby potentially the development of diabetes, we silenced the expression of three selected genes; *Gpx7*, *Gstt1* and *Snx19*, in clonal β-cells. These genes were selected based on their potential role in diabetes and islet function [47], [49]–[51] and because they showed both differential DNA methylation and gene expression between genotype groups in the mQTL and eQTL analyses (**Table S2 and Table S5**). One representative mQTL and eQTL for *GPX7,*, *GSTT1* and *SNX19*, respectively, is presented in **Figure 5A–C**. Moreover, *GPX7* and *GSTT1 * belong to the genes that were enriched in the glutathione metabolism KEGG pathway of significant *cis*-eQTLs. The knock-down experiments were performed to establish if identified genes in our mQTL analysis have a biological function in pancreatic β-cells. While both *GPX7* and *GSTT1* encode proteins that are known to protect against oxidative stress [48], [52], [53], sortin nexin 19, encoded by *SNX19*, may put cells into a pre-apoptotic state [50]. We therefore studied cell number and cell death signaling, measured as caspase-3/7 activities, under control and lipotoxic stress conditions when silencing selected candidate genes in clonal β-cells. The expression level of *Gpx7*, *Gstt1* and *Snx19* respectively, was significantly reduced in the siRNA knockdown experiments (P<0.05, **Figure 5D**). Interestingly, both under control and lipotoxic conditions, we found increased caspase-3/7 activities in β-cells with silenced *Gpx7* or *Gstt1* expression compared to negative control siRNA transfected (siNC) β-cells (P<0.05, **Figure 5E**). Moreover, when crystal violet staining was used to measure β-cell number, knockdown of *Snx19* resulted in increased cell number compared to negative control cells under both normal and lipotoxic conditions (P<0.05, **Figure 5F**).

### Associations of identified mQTLs with insulin secretion in human pancreatic islets

Pancreatic islets play a major role in controlling whole-body glucose-homeostasis through secreting insulin in response to elevated blood glucose levels and other fuels. To further examine phenotypic outcomes of significant mQTLs in human pancreatic islets, significant *cis* and *trans* mQTL-SNPs were related to glucose-stimulated insulin secretion from human islets *in vitro*. Out of the identified *cis*-mQTL-SNPs, 1,843 (5.0%) SNPs were associated with glucose-stimulated insulin secretion *in vitro* (P<0.05) (**Table S11**). Moreover, seven of the *cis*-mQTL-SNPs associated with insulin secretion were also identified in the *cis*-eQTL analysis including the *GPX7* and *HLA* genes (**Table S5**). Additionally, out of the identified *trans*-mQTL-SNPs, 90 (6.1%) SNPs were associated with glucose-stimulated insulin secretion in human islets (**Table S12**). We next used CIT [32] to examine if relationships between genotypes and phenotype (insulin secretion) were potentially mediated through DNA methylation of CpG sites in the significant mQTLs. In this CIT approach, we consider genotypes of SNPs identified in the mQTL analysis as causal factors (G), DNA methylation of CpG sites identified in mQTL analysis as potential mediators (M) and islet insulin secretion as a phenotypic outcome (I). The possible relationships between these three factors are shown in **Figure S8A**. Significant SNP-CpG pairs from the mQTL analysis where mQTL-SNPs also show association with insulin secretion were included in the CIT (**Figure S8B**). The CIT analysis of *cis*-mQTLs identified 14 (0.5%) SNP-CpG pairs consisting of 10 unique SNPs and 8 unique CpGs that were called as causal (causal hypothesis P-value<0.05; *nothing hold for FDR with Q-value<0.05*) and represent potential methylation-mediated relationships between mQTL-SNPs and insulin secretion (**Figure S8B** and **Table S13**). One identified mQTL, where methylation potentially mediates the causal association between the SNP and islet insulin secretion, was annotated to *PTPRN2* (also known as *IA-2β* or in rodents as *phogrin*) (**Table S13**). Interestingly, the *PTPRN2* gene encodes a protein that is an autoantigen in type 1 diabetes [54], [55]. When performing the CIT analysis of *trans*-mQTLs, no SNP-CpG pairs were found to show a causal relationship with islet insulin secretion (**Figure S8B**).

### Identified mQTLs/eQTLs in human pancreatic islets capture reported diabetes SNPs

Previous GWAS have identified SNPs associated with an increased risk of diabetes or diabetes related traits [15], [20], [56]. Nevertheless the molecular understanding of how these SNPs contribute to disease is still limited. To examine if previously reported diabetes SNPs may affect DNA methylation and/or gene expression in human pancreatic islets, a key tissue in the pathogenesis of diabetes, they were checked for overlap with the identified mQTLs/eQTLs in the present study.

The GWAS catalog (www.genome.gov/gwastudies, accessed March 2013) [57] was used to find SNPs reported to be associated with diabetes. In total, 317 SNPs were identified showing associations (P<10^−6^) with type 1 diabetes, type 2 diabetes or related traits (glucose-, insulin- and proinsulin traits). To get better reference coverage of these SNPs a proxy search using SNAP [58] was performed, giving 5,448 SNPs in LD (r^2^>0.8) with the reported diabetes SNPs. This dataset was then used to check for any overlap with the identified SNPs in the mQTL/eQTL analyses of human islets.

**Figure 6:**
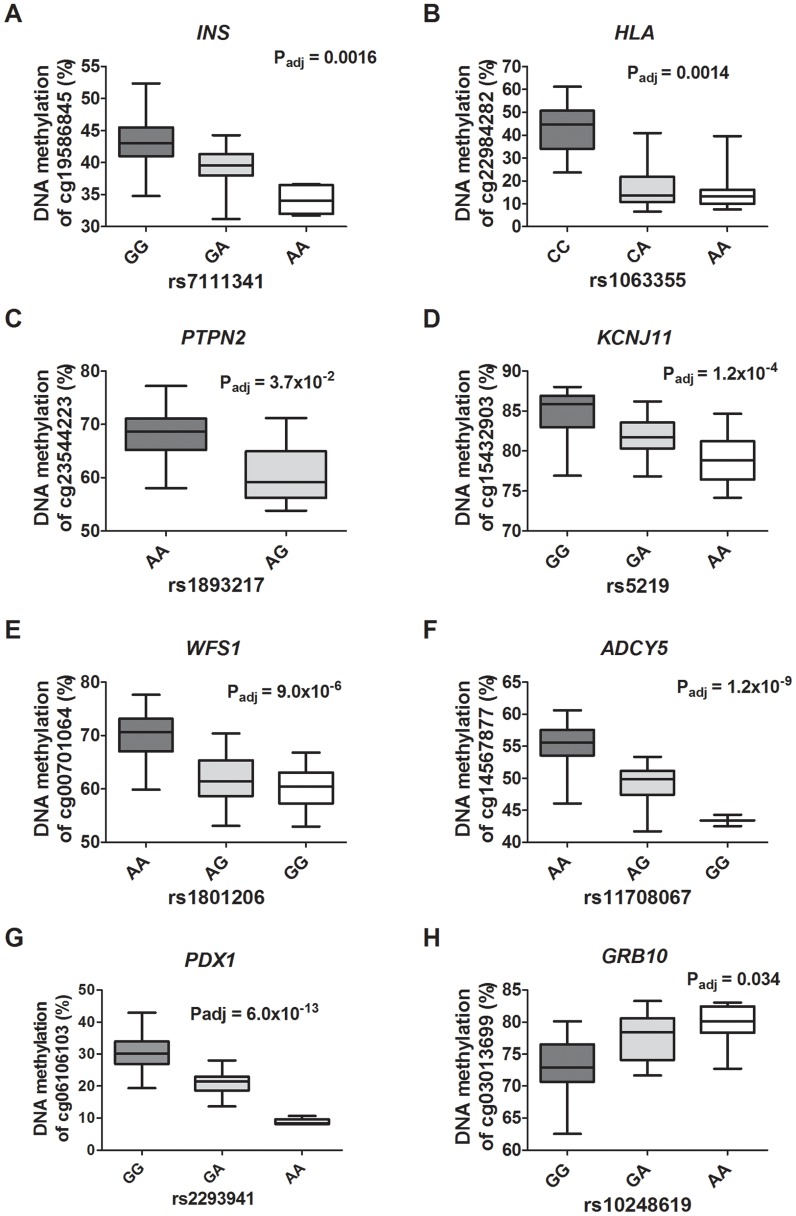
Diabetes SNPs reported by GWAS associate with DNA methylation in human pancreatic islets. Depiction of some identified associations between SNP and DNA methylation in islets of reported type 1 diabetes loci: (**A**) *INS*, (**B**) *HLA* and (**C**) *PTPN2*; type 2 diabetes loci: (**D**) *KCNJ11*, (**E**) *WFS1* and (**F**) *ADCY5*; and glucose-trait loci: (**G**) *PDX1* and (**H**) *GRB10*. P-values adjusted for multiple testing. *HLA* rs1063355 and *WFS1* rs1801216 were identified through proxy search and are in linkage with the GWAS reported diabetes SNPs *HLA* rs9272346 and *WFS1* rs1801214, respectively.

In the overlap, 32 out of 317 (10.7%) reported diabetes SNPs were found to match directly or through a proxy with the identified *cis*-mQTL-SNPs, consisting of SNPs associated with type 1 diabetes (n = 12), type 2 diabetes (n = 12), fasting-plasma glucose (n = 4; 1 SNP overlapping with type 2 diabetes), 2 hour glucose challenge (n = 1), insulin response (n = 2) and proinsulin (n = 2) (**Figure 6A–H** **; Table S14**). Moreover, one diabetes associated SNP (rs9272346 *HLA-DQA1*, P_T1D_<10^−128^) was found through the proxy search to overlap with a *cis*-mQTL-SNP (rs1063355 *HLA-DQB1*, R^2^ = 0.87) (**Figure 6B**) that showed association with mRNA expression in the human islets (**Table S5**). Identified *trans*-mQTL-SNPs were not found to overlap with reported diabetes SNPs identified through GWAS.

Identified mQTL-SNPs were also checked for overlap with publicly available consortium data of type 2 diabetes associations from DIAGRAMv3 GWAS meta-analysis [59] and for glycemic traits association from MAGIC investigators [60]–[64]. Significant mQTL-SNPs overlapping with SNPs showing associations with type 2 diabetes (P<0.05 in DIAGRAM) or with glucose, insulin and proinsulin traits (P<0.05 in MAGIC) are presented in **Table S15** and **Table S16**, *cis*- and *trans*-mQTL-SNPs respectively. These include SNPs annotated to the *KIF11-HHEX-IDE* region, *WFS1, ADCY5, KCNJ11, FADS1, SIRT2* and *SNX19*.

As an evaluation of the number of islet mQTL-SNPs also reported to be diabetes associated SNPs in GWAS, we further checked for overlap between mQTL-SNPs identified in human islets and SNPs associated with breast cancer, stroke and hypothyroidism; diseases not relevant for our targeted tissue of pancreatic islets. In total, there were 63 reported SNPs associated with breast cancer, 18 SNPs associated with stroke and 20 SNPs associated with hypothyroidism in the GWAS catalog with P<10^−6^ (accessed March 2013). Out of these, four breast cancer SNPs, one SNP associated with stroke and no hypothyroidism SNPs could be identified directly or through a proxy SNP as *cis*-mQTLs in human pancreatic islets. However, the SNPs associated with the additional traits were neither identified in the *trans*-mQTL analysis nor in the eQTL analyses of human islets.

### Associations between DNA methylation and mRNA expression in human pancreatic islets

**Figure 7:**
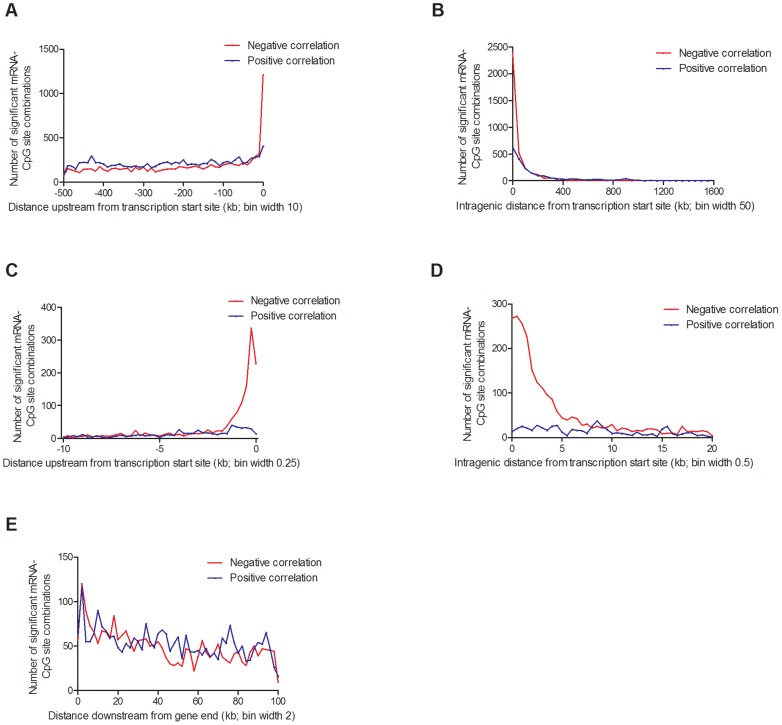
Distribution of CpG sites significantly associated with one or more mRNA transcripts, separated based on negative or positive correlations. (**A**) 20,376 combinations in the region 0–500 kb upstream of transcription start site and (**B**) 5,718 intragenic combinations. Negative correlations were enriched in the region surrounding the transcription start site, both (**C**) upstream and (**D**) downstream. (**E**) 5,221 combinations 0–100 kb downstream of the gene. Associations corrected for multiple testing using false discovery rate at 5% (Q<0.05).

Depending on the genomic location of a CpG site, DNA methylation may regulate gene transcription in several different ways [65], [66]. Nevertheless, the association between DNA methylation and gene expression throughout the human genome remains poorly described. To test if DNA methylation is directly associated with gene expression in human pancreatic islets, we performed a linear regression between individual mRNA transcripts and DNA methylation of CpG sites in *cis* (500 kb up- and 100 kb downstream of respective gene), including age, gender, BMI, HbA1c, islet purity, days in culture and batch as covariates. We found significant associations between DNA methylation and mRNA expression for 31,315 combinations (FDR<5%), consisting of 22,773 unique CpG sites (4.9% of tested CpG sites) and 5,377 unique mRNA transcripts (19.6% of tested mRNA transcripts), which are annotated to 4,876 genes. Out of these, CpG sites in 20,376 combinations (65.1%) were located in the region 0–500 kb upstream of a transcription start site, CpG sites in 5,718 combinations (18.3%) were intragenic, and CpG sites in 5,221 combinations (16.7%) were located 0–100 kb downstream of a gene (**Figure 7**). For CpGs upstream from a transcription start site, 9,436 combinations (46.3%) showed negative and 10,940 combinations (53.7%) showed positive correlations between DNA methylation and mRNA expression (**Figure 7A**). For intragenic CpGs, we found 3,694 (64.6%) negative and 2,024 (35.4%) positive correlations (**Figure 7B**). Interestingly, negative correlations were enriched for CpGs in the region close to the transcription start site (**Figure 7C–D**). For example, for CpGs in the region 1 kb upstream to 1 kb downstream from the transcription start site, 90% of the correlations between DNA methylation and mRNA expression were negative. For CpGs downstream of the gene, we found negative correlations for 2,499 combinations (47.9%) and positive correlations for 2,722 combinations (52.1%) (**Figure 7E**).

In addition, we looked for any overlap between significant mQTL/eQTL results and direct associations between DNA methylation and mRNA expression. Thereby, we extracted and paired CpG sites and mRNA transcripts that were significantly affected by the same SNPs in the mQTL/eQTL analyses, which resulted in identification of 410 unique CpG-mRNA transcript pairs. Out of these, 287 (70%) also showed a significant direct association between DNA methylation and mRNA levels, where 164 (57.1%) CpG-mRNA transcript pairs showed negative correlations and 123 (42.9%) showed positive correlations (**Table S17**). Of note, for all three genes selected for functional follow-up experiments based on both significant mQTL and eQTL results (**Figure 5**), DNA methylation was directly associated with gene expression, e.g. DNA methylation of 8 CpG sites within or around *GSTT1* showed the most significant correlations with mRNA expression of *GSTT1* (P_adj_<9.9×10^−13^) (**Table S17**). Additionally, DNA methylation within or around *GPX7* and *SNX19* was directly associated with mRNA expression of respective gene (**Table S17**).

### Biological validation and replication of mQTL and eQTL data

**Figure 8:**
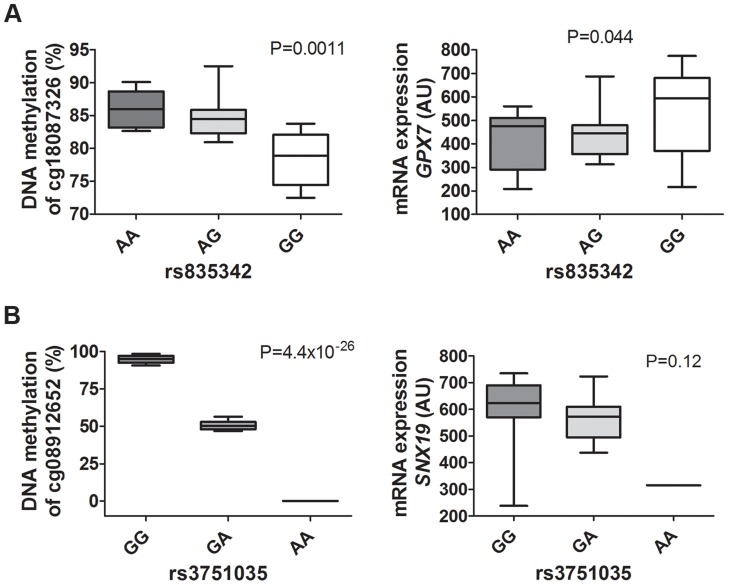
mQTLs/eQTLs of *GPX7* and *SNX19* identified in the genome-wide analysis were biologically validated in pancreatic islets from a different set of human donors. Biological validation of associations for (**A**) *GPX7* rs835342 with DNA methylation of cg18087326 as well as with mRNA expression of *GPX7* and (**B**) *SNX19* rs3751035 with DNA methylation of cg08912652 as well as with mRNA expression of *SNX19* in a set of human pancreatic islets from donors (n = 37) not included in the genome-wide mQTL/eQTL analysis. DNA methylation was analyzed using Pyrosequencing and mRNA expression using Affymetrix microarray. Data are presented as Box and Whisker plots with P-values.

To biologically validate our findings from the genome-wide mQTL analysis and the eQTL analysis, we analyzed DNA methylation with Pyrosequencing and mRNA expression of two selected genes (*GPX7* and *SNX19*) in pancreatic islets from a different set of human donors than the ones used for the mQTL/eQTL analyses. The characteristics of the 37 islet donors used for biological validation can be found in **Table S18**. Importantly, our mQTL/eQTL data could be biologically validated in the new set of islets (**Figure 8A–B** **,** **Figure 5** **, Table S2 and Table S5**). We found significant differences in methylation and expression between genotype groups which were in the same direction as the genome-wide mQTL/eQTL analysis. Of note, for validation of *SNX19* expression, there was only expression data available from one carrier of the rare variant and the association did not reach significance, P = 0.12 (**Figure 8B**). It should also be noted that we were able to validate significant mQTL data detected with an Infinium probe that contains a SNP by the use of Pyrosequencing (**Figure 5C** and **Figure 8B**), i.e., there is a SNP (rs4402303, C/T) located in the *SNX19* methylation probe (cg08912652, **Table S2**), which either introduces or removes a CpG site and this SNP is in full LD with our significant mQTL SNP (rs3751035; D′ = 1, r^2^ = 1 based on 1000 Genomes project, CEU population panel, distance between SNPs = 5.7 kb).

We further examined whether our significant islet *cis*-mQTLs (presented in **Table S2**) were identified in previous reported mQTL studies from other human tissues [8], [9], [12]–[14]. Here, we tested for the overlap of CpG sites in significant mQTLs in our study and previously reported mQTL studies. For example, we found that ∼33% of identified CpG sites in significant *cis*-mQTLs in our human islet study were also identified in significant *cis*-mQTLs in adipose tissue [12]. The numbers of CpG sites in significant *cis*-mQTLs in our human islets study that could be replicated in previously published human mQTL studies are presented in **Table S19**. Significant *cis*-mQTLs identified in human pancreatic islets and not replicated in other human tissues may be islet specific. In total, we found 6,898 CpG sites in significant *cis*-mQTLs annotated to 3,241 unique genes in our islet mQTL analysis that cannot be replicated in any of the previously published human mQTL studies used in the overlap analysis [8], [9], [12]–[14]. To look for potential biological relevance of significant *cis*-mQTLs only identified in human islets, we performed KEGG pathway analysis of this subset of 3,241 unique genes (**Table S20**). However, we cannot rule out that unequal filtering and inclusion criteria of CpG probes, different significance thresholds for calling mQTL hits and various *cis* windows together with other factors may influence the replication of our islet mQTLs in previously published mQTL studies in other human tissues.

### Associations between imputed genotype data and DNA methylation in human pancreatic islets

To generate a reference map of mQTL data in human pancreatic islets, we finally imputed autosomal genotype data generated with the Human OmniExpress BeadChip for the 89 islet donors to the 1000 Genomes phase 1, version 3 reference panel. We then associated imputed autosomal genotype data, including 6,544,062 SNPs, with DNA methylation data of 468,787 CpG sites from islets of 89 human donors. Based on significance thresholds of 4.9×10^−10^ and 2.5×10^−13^ in the *cis*- and *trans*-mQTL analyses, respectively, we found 978,128 SNP-CpG pairs in *cis* (**Table 5** and **Table S21**) and 59,529 SNP-CpG pairs in *trans* (**Table 5** and **Table S22**) showing significant associations between genotypes and DNA methylation levels. These 978,128 *cis*-SNP-CpG pairs consist of 494,642 unique SNPs (7.6% of tested SNPs) and 14,308 unique CpG sites (3.1% of tested CpG sites), which are annotated to 5,160 unique genes (**Table 5** and **Table S21**). Moreover, the 59,529 *trans*-SNP-CpG pairs consist of 34,351 unique SNPs (0.5% of tested SNPs) and 545 unique CpG sites (0.1% of tested CpG sites), which are annotated to 352 unique genes (**Table 5** and **Table S22**). Of note, only 2,573 new CpG sites were discovered in the *cis*-mQTL analysis of imputed genotype data compared with the *cis*-mQTL analysis of directly genotyped SNP data (**Table 1**/**Table S2** and **Table 5**/**Table S21**). Additionally, we discovered 162 new CpG sites in the *trans*-mQTL analysis of imputed genotype data compared with the analysis of directly genotyped SNP data (**Table 1**/**Table S3** and **Table 5**/**Table S22**). The mQTL analysis of imputed genotype data identified all significant SNP-CpG pairs presented in **Table S2** and **Table S3**. The modest increase in discovered CpG sites and unique genes in the mQTL analysis of imputed SNPs is most likely due to a dependency in imputed and directly genotyped SNP data as the directly genotyped SNP data generated with the Human OmniExpress BeadChip was used for imputation.

**Table 5:** Number of significant mQTL results in human pancreatic islets when including imputed genotyped data.

	*cis* -mQTL	*trans* -mQTL
SNP-CpG pairs	978,128	59,529
Unique SNPs	494,642	34,351
Unique CpG sites	14,308	545
Unique genes	5,160	352

Significance threshold <0.05 after correction for multiple testing.

Correction value *cis* = 102,307,720.

Correction value *trans* = 200,388,516,440.

## Discussion

It is well established that genetic and epigenetic variation contributes to the development of numerous diseases, including diabetes [40], [56], [65], [67]–[73]. While most studies have investigated genetic and epigenetic mechanisms independent of each other, they may interact and together affect biological processes and susceptibility to disease. Here, we perform the first mQTL analysis in human pancreatic islets targeting DNA methylation of ∼99% of RefSeq genes and most genomic regions in the human genome. The present study gives new insights on how genetic and epigenetic factors can interact in humans and provides a detailed map of genetic loci affecting the genome-wide DNA methylation pattern in human pancreatic islets.

Pancreatic β-cells secrete insulin in proportion to extracellular glucose concentrations and thereby contribute to whole-body glucose-homeostasis. Deficient insulin secretion, giving rise to chronically elevated blood glucose levels, is a hallmark of diabetes mellitus. Recent GWAS have identified SNPs associated with an increased risk of both type 1 diabetes [15]–[17], [19], [21], [22], [24] and type 2 diabetes [25]–[28]. Interestingly, many of these SNPs seem to affect pancreatic islet function, autoimmunity and inflammation [15], [23], [74]–[79]. However, SNPs identified by GWAS only explain a small part of the estimated heritability of type 2 diabetes based on family studies [31], suggesting that there are additional genetic factors left to be discovered. SNPs that are carriers for inheritance of DNA methylation may explain some of the missing heritability of complex diseases. In the present study, we found that SNPs associated with DNA methylation, mRNA expression and insulin secretion in human pancreatic islets also showed nominal associations with type 2 diabetes as reported by the DIAGRAM consortium [59] and with glucose/insulin traits as reported by MAGIC investigators [60]–[64]. It is possible that some of the overlapping SNPs have escaped detection to disease phenotypes in previous GWAS and that association to diabetes can only be significantly detected if the degree of DNA methylation in cases and controls is taken into account. However, other cohorts than the one used in this study will be needed to test this. Environmental factors can change the degree of DNA methylation and may thereby control phenotype transmission [67], [71], [72], [80]–[82]. Effects of SNPs that interact with DNA methylation levels may thereby change under different environmental conditions, which could affect their impact on disease risk [70]. This may be one explanation for gene-environment interactions.

The majority of reported loci that predispose to diabetes seem to act through insulin secretion defects from pancreatic islets [83]–[85]. However, the molecular mechanisms of how most of these SNPs affect their target gene or phenotypic outcome remain unknown. In the present study, we found that several SNPs identified in GWAS to associate with type 1 diabetes (e.g. *PTPN2* [15], *INS* [15] and *HLA* [86]), type 2 diabetes (e.g. *ADCY5* [60], [64], [87] and *KCNJ11* [25], [26], [29], [79]) and glucose-traits (*GRB10* [64] and *PDX1* [64]) were also associated with differential DNA methylation between genotype groups in human pancreatic islets. In particular we found an enrichment of significant mQTLs in the HLA region on chromosome 6p21, which possess the strongest genetic determinant for type 1 diabetes [23] and predisposition to autoimmunity [78]. In total, 55% of the CpG sites in significant *cis*-mQTLs on chromosome 6 were located within the HLA gene region (Chr6:29.570.005–33.377.701 - human genome build 37) and the enrichment cannot be explained by the distribution of analyzed sites on the array. A non-HLA gene, *PTPN2*, known to affect the risk of type 1 diabetes and Crohn's disease was also identified in the mQTL analysis of human islets [15], [74]. *PTPN2* encodes a non-receptor type protein member of the tyrosine phosphate family and is expressed in β-cells where it has been shown to be involved in cytokine-induced apoptosis [75], [88]. We also found significant mQTLs in the *PDX1* and *INS* (insulin) genes. PDX1 (pancreatic duodenal homeobox 1) is a transcription factor involved in pancreas development and function [89], [90]. The *PDX1* gene is also expressed in β-cells of the mature pancreas, where it plays a role in glucose-dependent regulation of insulin gene expression and insulin secretion. Recent studies from our group show that increased DNA methylation may reduce expression of *PDX1* and *INS* in diabetic islets and contribute to the development of the disease [40], [68], [91]. Altered DNA methylation levels in human pancreatic islets based on genotype may be a molecular mechanism through which diabetes associated SNPs contribute to the disease phenotype.

We recently showed that ∼50% of previously reported type 2 diabetes risk SNPs are so called CpG-SNPs that introduce or delete possible DNA methylation sites. These type 2 diabetes associated CpG-SNPs were significantly associated with altered DNA methylation, gene expression and islet hormone secretion in pancreatic islets from non-diabetic human donors [65]. In the present study, we also looked for associations between significant mQTL-SNPs and islet insulin secretion in our study cohort and we found numerous associations with P<0.05. However, the lack of available insulin secretion data measured in pancreatic islets *in vitro* in an independent cohort limits our possibility to replicate and strengthen our results. Nevertheless, our findings may provide interesting biological insights to the field of insulin secretion.

Further, in order to mathematically model the relationships between genotype, DNA methylation and a phenotype (mRNA expression and insulin secretion), we performed CIT analysis [32]. While the CIT for mRNA expression remained significant after correction for multiple testing, the CIT for insulin secretion did not stand correction for multiple testing. Interestingly, we found that genetic associations with mRNA expression of genes located in the *HLA* region and of genes involved in glutathione metabolism were potentially mediated through DNA methylation. Both the *HLA* gene region and the glutathione genes have been genetically linked to type 1 diabetes and are suggested to play a biological role in islet function [37], [47]. Our data also suggest that DNA methylation of a CpG site within *PTPRN2* is the potential mediator of the association between a SNP in the same gene and islet insulin secretion. The gene product of *PTPRN2* (also known as *IA-2β* or in rodents as *phogrin*) is a receptor type of the protein tyrosine phosphatase family known to be a major islet autoantigen in type 1 diabetes [54], [55]. Expression of the *PTPRN2* gene product in pancreatic islets is shown to have important biological β-cell functions and is involved in the regulation of insulin secretion [92]–[95]. Together with the mQTL findings in e.g. *HLA* genes and *PTPN2*, our results highlight that future studies may need to integrate genetics and epigenetics in order to clarify how candidate genes for type 1 diabetes contribute to the disease. To our knowledge, only two previous studies have applied a CIT approach to model the interacting relationship between genotype and DNA methylation on the effect of a human phenotype [13], [96]. In line with the study by Liu et al. that found ten differentially methylated positions in blood that mediate genetic risk in rheumatoid arthritis [96], we found in the present study 14 differentially methylated positions in human islets that act as potential mediators of genetic associations with mRNA expression. Since the CIT analyses are based on hypotheses that mathematically model the causal relationships of interactions between genetics and epigenetics on phenotypes, we cannot rule out the fact that confounding factors not coped for in the models may influence the suggested calls of causality. Although independent studies need to verify the modelled relationships, we will emphasize that the study approach previously addressed by Liu et al [96] and Gutierrez-Arcelus et al [13] and applied here reveals novel interesting information about molecular interactions between genetics and epigenetics, and may pose new questions about disease causality.

Functional *in vitro* follow-up studies in β-cells of selected genes, based on our mQTL/eQTL findings, showed that decreased expression of *Gpx7* and *Gstt1* significantly affects caspase activity and decreased expression of *Snx19* significantly affects cell number. These functional experiments were performed to test if any of the identified genes in the mQTL/eQTL analyses have a biological role in β-cells. Importantly, we could also biologically validate our mQTL/eQTL results for *GPX7* and *SNX19* in a different set of islets than the ones included in the genome-wide analysis. Together, our data propose a model where altered DNA methylation and expression of these genes in human islets based on genotype may influence *in vivo* islet β-cell number and thereby diabetes risk. Interestingly, *GPX7* (glutathione peroxidase 7), and *GSTT1* (glutathione S-transferase theta 1), are involved in glutathione metabolism, a pathway we found to be enriched among differentially expressed genes in the eQTL analysis of the human islets, and known to have cell protective functions against oxidative stress [48], [49], [52]. Moreover, the protein encoded by *SNX19* (sortin nexin 19) has been shown to interact with the islet autoantigen IA-2 and put cells into a pre-apoptotic state [50]. Here, we identified numerous mQTL loci that affect the expression of these genes. Interestingly, some of these loci were also nominally associated with glucose traits in analyses by MAGIC investigators [60]–[64]. Together, our functional data provide novel biological insights in the regulation of β-cell function.

Additionally, the genes covering significant *cis*-mQTLs were enriched in a total of 11 KEGG pathways. These include 9 KEGG pathways relevant to pancreatic islet function, e.g. type 1 diabetes [88], [97], cell adhesion molecules [98], extracellular-receptor matrix (ECM) interaction [99] and folate biosynthesis [100]. It should be noted, however, that many individual genes are included in multiple KEGG pathways and the significant pathways that do not seem to be relevant for pancreatic islet function, such as viral myocarditis, contain numerous individual genes with important roles in pancreatic islets e.g. *CASP3*, *CAV1* and *HLA*-genes [101]–[103]. Moreover, for the analyses of genes covering significant *cis*-eQTLs and *trans*-mQTLs, all of the identified KEGG pathways were relevant to pancreatic islet function [49], [104]–[111].

This study is to our knowledge the first to perform both *cis* and *trans* mQTL analyses of DNA methylation data generated with the Infinium HumanMethylation450 BeadChip. Our aim was to select a *cis* distance that illustrates the overall distribution of significant *cis*-mQTLs. Before selecting 500 kb as our *cis* distance, we performed a preliminary mQTL analysis where we used 1 Mb as the *cis* distance. However, based on the small number of significant SNPs identified in the *cis* window between 501 kb and 1 Mb (e.g. only 1.43% of significant mQTL-SNPs were located in the 501–1000 kb window, while 98.57% of significant mQTL-SNPs were located within the 0–500 kb window), we reduced the *cis* distance to 500 kb. Quon et al have previously tried to find an optimal window size for inclusion of *cis* acting SNPs for mQTL analysis of methylation data from the human brain generated with the Infinium HumanMethylation27 BeadChip [112]. Here, they propose that using a too large or too small *cis* window dramatically reduced the number of identified heritable loci. However, it should be noted that the optimal *cis* distance may vary in different tissues and cell types.

In our mQTL analysis, we took advantage of the sampling of DNA methylation across the genome to explore distribution of mQTLs in genomic regions based on relation to the nearest gene or in relation to the nearest CpG island. Interestingly, based on Illumina's annotations, we found an enrichment of significant *cis*-mQTLs in the gene body and intergenic regions, as well as in northern- and southern shores, southern shelf and open sea. Additionally, we found less significant *cis*-mQTLs than expected in CpG islands. Most of the previous mQTL analyses, which mainly cover DNA methylation data in CpG islands of promoter regions, have subsequently not been able to describe the genomic location of significant mQTLs [8], [9], [113]. However, our study suggests that DNA methylation in more CpG-depleted regions to a larger extent is regulated by genetic factors. These results confirm previous efforts from our group and others [12], [72], [91], [114]. Interestingly, a very recent study from our group shows that differentially methylated CpG sites in pancreatic islets from patients with type 2 diabetes compared to non-diabetic donors are also overrepresented in intergenic regions and the open sea while underrepresented in CpG islands [91]. These results are also in line with a previous global analysis of DNA methylation in adipose tissue from twins using the Illumina 450 K chip, where they showed that high variability of DNA methylation in the gene body and intergenic regions across individuals can be explained by regulation of genetic factors [12]. We further took advantage of the published mQTL data in adipose tissue from Grundberg et al [12] and analysed if the genomic distribution of their significant *cis*-mQTLs show a similar pattern to the findings in our study. Confirmative, significant *cis*-mQTLs in human adipose tissue were overrepresented in the intergenic region, the gene body, the open sea as well as the shore and shelf regions, while underrepresented in regions close to the TSS and CpG island regions based on Illumina's annotations. In agreement with the data in the present study, we have previously found that CpG sites with significantly altered methylation in human adipose tissue after an exercise intervention or based on type 2 diabetes are enriched in the gene body, intergenic region and open sea, while underrepresented in the CpG island region [72], [114]. Together, our genome-wide data point to a direction that variable CpG sites in the human genome are more frequently located outside CpG rich regions. Moreover, the role of DNA methylation seems to vary in context between different genomic elements, and although the function of DNA methylation in gene body and enhancer regions is less well studied compared to promoter methylation, DNA methylation in these genomic regions seems to be crucial for biological function and cell regulation [40]–[43], [66]. It is possible that CpG sites annotated to intergenic regions in our study overlap with enhancer regions and thereby involve distal gene regulatory elements. Moreover, CpG sites located within gene bodies or non-coding regions of a gene may overlap with enhancer elements for another distant gene [115]. Additionally, it has also been suggested that the relationship between gene body DNA methylation and expression is bell shaped and varies depending on the transcriptional activity of the gene, e.g. that high levels of gene body methylation are observed in genes with moderate expression levels while low levels of gene body methylation are observed in genes with low and high expression [116].

Although our mQTL analysis was performed in pancreatic islets of to date the largest cohort of human islet donors, our statistical power is limited compared to large genetic population studies. Nevertheless, after correction for multiple testing, we identified ∼67,000 significant SNP-CpG pairs in human islets which demonstrate a strong interaction between genetic and epigenetic mechanisms. It may seem surprising that we found such a large number of significant associations between SNPs and DNA methylation in human islets from 89 donors after correcting for multiple testing (e.g. we corrected for 102,307,720 tests in *cis*). However, our mQTL data in human islets are in line with previous mQTL analyses performed in human brain samples, where ∼8,000–12,000 significant SNP-CpG pairs were identified when DNA methylation of only ∼27,000 CpG sites was analyzed in approximately 100–150 samples [8], [9]. One should also keep in mind that ∼28% of common SNPs in the human genome either introduce or remove a CpG site [117]. These so called CpG-SNPs can have very strong effects on DNA methylation in human tissues [65]. They have also been shown to be biologically relevant [70], [118]–[125]. Altering the binding of certain proteins is one possible mechanism through which methylation in CpG-SNPs can affect gene expression. For example, a recent study showed that DNA methylation of a CpG site created by the G allele of a CpG-SNP located in the 5′UTR of the *GDF5* gene altered the binding affinity for SP1 and SP3 repressor proteins which have a higher affinity to the unmethylated allele and this lead to an expression imbalance between both alleles [118]. Interestingly, another study identified a variant associated with alcohol dependence that introduces a CpG site in *PDYN*. Even though carriers of the T risk allele had the highest binding affinity for a protein that regulates *PDYN* expression positively the researchers found that increased DNA methylation of the non-risk C allele increased its binding affinity for this protein more than the non-methylated C allele but still less than the risk T allele. Methylation of the C allele resulted in increased PDYN expression and made it act similar to the risk allele, and it is possible that the increase in DNA methylation may be a consequence of alcohol consumption [119]. Additionally, our group has previously reported a CpG-SNP in the promoter of *NDUFB6* that shows increased DNA methylation in skeletal muscle from elderly but not young subjects which resulted in reduced *NDUFB6* expression and insulin-stimulated glucose uptake only in the elderly subjects [70]. This demonstrates that the phenotypic outcome of a CpG-SNP can result not only from genotypic differences but that even carriers of the same genotype can have a different phenotype depending on the degree of DNA methylation of the SNP site which can be influenced by lifestyle and age. Interestingly, a CpG-SNP in the promoter of *CYP17A1* is associated with Oligoasthenoteratozoospermia and testosterone levels in infertile males and the degree of methylation in the SNP site was high in colon and stomach tissue while low in testis, kidney and adrenal gland [120]. The tissue specific DNA methylation pattern within the CpG-SNP site of *CYP17A1* was further associated with high *CYP17A1* expression in tissues with low methylation in the SNP site. In addition, intragenic CpG-SNPs can influence transcription elongation positively or negatively through alternative promoters or noncoding transcripts [121]–[123]. Methylation of a CpG-SNP can also play a role in the regulation of splicing by helping the splicing machinery to identify exons [124] or by affecting recombination rates [125]. Together, these studies support key biological functions of differential DNA methylation due to CpG-SNPs.

It should also be noted that previous human case-control studies [73], [91], [126] and human intervention studies [71], [72], [80] have identified quite a large number of significant differences in DNA methylation in cohorts with less than 100 samples. Together, these studies demonstrate that both genetic and environmental factors can have strong effects on the human methylome and that a large number of significant differences in methylation can be found in modest sample-sizes.

Epigenetic modifications are involved in the regulation of gene transcription [67]. However, no previous study has to our knowledge related DNA methylation data generated with the HumanMethylation450 BeadChip to genome-wide expression data. Here, we found direct associations between DNA methylation of 22,773 CpG sites and mRNA expression of 4,876 genes in human islets. Interestingly, ∼2/3 of the CpG sites that showed significant associations with mRNA expression were located upstream of a transcription start site. Additionally, 90% of the associations were negative when CpG sites were located in the region 1 kb upstream to 1 kb downstream of the transcription start site. These data are in line with our previous studies where we have shown that DNA methylation in promoter regions close to the transcription start site has direct negative effects on the transcriptional activity using luciferase assays [71], [72]. While methylation close to a transcription start site is known to block initiation of transcription, methylation in the gene body might contribute to transcriptional elongation [66]. In the present study, 35.4% of the associations between gene expression and DNA methylation of intragenic CpGs were positive. Associations between expression and methylation of CpGs located downstream of genes have not been studied in human genome-wide data. We found direct associations between expression and methylation of CpGs located downstream of genes, where 47.9% of the associations were negative. However, it remains to be tested if methylation downstream of a gene affects the transcriptional machinery. Additionally, for ∼70% of identified mQTLs affecting gene expression there was also a direct association between DNA methylation and gene expression in human islets, suggesting that altered DNA methylation in the mQTL has a direct impact on gene expression. The CIT further supported this hypothesis. Although, these novel data improve our understanding of the associations between DNA methylation and gene expression throughout the genome, additional studies are needed to examine if the genome-wide association-pattern between methylation and expression is tissue specific or general for multiple human tissues.

The key biological findings of our study include; i) the identification of a large number of SNPs with strong effects on DNA methylation in human pancreatic islets; ii) the discovery of SNPs previously known to affect diabetes and its related traits that affect DNA methylation in human pancreatic islets; iii) the first demonstration of how SNPs can mediate their effects on gene expression via altered DNA methylation in human pancreatic islets; iv) the strong genetic regulation of DNA methylation in genomic regions with low CpG density; and v) the illustration of how the genome-wide DNA methylation pattern correlates directly with gene expression in human pancreatic islets. Impaired insulin secretion is a hallmark of diabetes. Understanding gene regulation in human pancreatic islets is therefore essential for creating a full picture of diabetes and for optimal drug development. As the prevalence of diabetes is rapidly increasing worldwide, the need for new treatment strategies for diabetic patients is growing. New treatments may include epigenetic editing, where selected genes are targeted [127]. The results from our study may then be used to identify target genes for epigenetic editing. Additionally, a growing body of literature proposes that new therapeutic treatments for diabetes may target epigenetic mechanisms e.g. enzymes responsible for altering the epigenetic pattern in target tissues for the disease [128], [129]. Importantly, our study shows that subjects at risk for diabetes, by carrying genetic risk variants for the disease, have altered DNA methylation in their pancreatic islets, and future therapeutics targeting epigenetic modifications may potentially reduce the risk for diabetes in these subjects.

In conclusion, we describe for the first time genome-wide interactions between genetic and epigenetic variation in human pancreatic islets. We show that interactions of these regulatory mechanisms can influence islet mRNA expression, islet function and potentially diabetes risk. Our results demonstrate the importance of considering epigenetics when studying the impact of genetic variation on phenotypic outcomes and human complex diseases. All together, these data can serve as a reference for future studies further dissecting the impact of genetic variation on epigenetic traits as well as for the understanding of epigenetic regulation of biological mechanisms.

## Methods

### Ethics statement

The pancreatic islet donor or her/his relatives had given their written or oral informed consent to donate organs for medical research upon admission to intensive care unit. All procedures were approved by ethics committees at Uppsala and Lund Universities.

### Sample information

Pancreatic islets from 89 human donors not diagnosed with diabetes mellitus were obtained from the Nordic Network for Islets Transplantation, Uppsala University, Sweden (**Table S1**). This islet cohort is a resource within the human tissue laboratory of Lund University Diabetes Center (http://www.ludc.med.lu.se/platforms/human-tissue-laboratory/) and data from this cohort has previously been described [91], [130]–[132]. Islets were prepared and cultured for 4.0±0.2 days prior to RNA and DNA isolation as previously described [68]. AllPrep DNA/RNA Mini Kit was used for islet DNA and RNA isolation (Qiagen GmbH, Hilden, Germany) and concentrations and quality were measured with NanoDrop ND-1000 spectrophotometer (NanoDrop Technologies, Wilmington, DE). Islet purity was 75±0.8% [133]. Glucose-stimulated insulin secretion was measured as stimulation index as previously described [134].

### Genotype data

Genome-wide genotyping was performed on DNA (200 ng) from 89 islet donors using the HumanOmniExpressBeadChip, which covers 731,412 SNPs and the iScan system (Illumina, Inc. CA) according to the Illumina protocol. Genotype calling was done with GenomeStudio software (Illumina). Quality control of genotype data was performed by PLINK software toolset [135]. All subjects passed the call rate threshold of >98% for inclusion. No gender discrepancy between the supplied donor information and the genotypic gender was detected. In population stratification analysis, no sample was highlighted as a population outlier supporting a homogenous ethnic make-up of the included islet donors. No donors were found to be related. SNP data were excluded from subsequent analysis based on following criteria's: call rate <98%; monomorphic SNPs; MAF<0.05; HWE<0.001; SNPs located on X and Y chromosomes due to bias of mixed gender population or with missing position. In total 574,553 SNPs passed quality control.

### DNA methylation analysis

Genome-wide DNA methylation profiling in pancreatic islets from the 89 human donors was assessed using the Infinium HumanMethylation450 BeadChip [39] (Illumina, Inc.), which analyzes DNA methylation in 482,421 CpG sites that cover 21,231 genes (99% of RefSeq genes) and all genomic regions [39]. DNA (500 ng) from pancreatic islets was bisulfite treated with the EZ DNA methylation kit (Zymo Research, Orange, CA) and used for analysis of DNA methylation with Infinium assay according to the standard protocol (User Guide part #15019519). BeadChips were imaged with Illumina iScan. All samples had an acceptable bisulfite conversion efficiency (intensity values >4000) [136] and passed quality control steps in GenomeStudio where built in control probes for staining, hybridization, extension and specificity were examined.

Subsequent analyses were performed using the lumi package from Bioconductor [137]. Methylation Beta-values were converted to M-values (M = log_2_(Beta/(1-Beta))) [138] and these were used for all statistical analysis. However, Beta-values were included in the final report for its biological interpretation (Beta = 2^M^/(2^M^+1)) [138]. Probes were then filtered and all CpG sites with a mean detection P-value<0.01 were considered detected and used for subsequent analysis. The methylation data were background corrected by subtracting the median intensities of built in negative controls and then normalized using quantile normalization [137], [139]. COMBAT was used to correct for batch effects [140]. While a strong batch effect could be identified before COMBAT was applied (P = 7.5×10^−6^ for correlation between batch and the 1^st^ component in a principal component analysis), there was no longer any identified batch effect after COMBAT (P>0.05 for the correlation between batch and first 10 principal components). After preprocessing of methylation data and exclusion of CpG sites located on X and Y chromosomes due to bias of mixed gender population, we obtained DNA methylation data for 468,787 CpG sites from human pancreatic islets. Probes reported to be cross-reactive (≥47 bases) or SNPs within underlying probe sequence, according to Chen et al. (2013) [33], are indicated in **Table S2** and **Table S3**. Based on the important role of CpG-SNPs on DNA methylation [65], probes with potential SNPs in the probe sequence were not filtered out from the mQTL analysis. The overall variability in DNA methylation from all 89 donors is illustrated in **Figure S1**.

### mRNA expression analysis

mRNA expression in pancreatic islets from 89 donors was analyzed genome-wide using the GeneChip Human Gene 1.0 ST array (Affymetrix, Santa Clara, CA) as previously described [133]. The array data was summarized and normalized using the Robust Multi-Array analysis method with the oligo package from Bioconductor. Gene transcripts with missing annotation or located on X and Y chromosomes were excluded from the dataset. COMBAT was used to correct for batch effects [140]. In total, mRNA expression of 27,391 transcripts was obtained for further analysis. The overall variability in mRNA expression from all 89 donors is illustrated in **Figure S3**.

### Methylation quantitative trait loci (mQTL) analysis

To test for associations between SNPs and DNA methylation, a linear regression model with biological covariates was used. In the linear model; DNA methylation values were used as the quantitative trait, SNP genotypes were encoded as 0, 1 or 2 according to the number of minor alleles, and the categorical variable gender as well as the continuous variables age, BMI, HbA1c, islet purity and islet culture days were included as covariates. The analysis was based on an additive genetic model. To distinguish between local (*cis*-) and distant (*trans*-) mQTLs, an arbitrary boundary with the maximum distance of 500 kb between SNPs and CpG sites were used to define *cis*-mQTLs. All other SNP-CpG pairs were considered as *trans*-mQTLs. The mQTL analysis was performed by using the R package Matrix eQTL [141]. P-values were adjusted with a correction value for multiple testing, which takes into consideration the dependency of linkage disequilibrium (LD) between SNPs by LD based pruning and thereby uses the number of independent tests. In the *cis*-analysis, LD based pruning of SNPs within a distance of 500 kb from a CpG site was performed by pairwise-tagging (r^2^<0.9) and the total sum of all tagSNPs connected to each CpG site was used as a correction value when correcting for multiple testing. LD calculations were performed using R trio package (http://www.bioconductor.org/packages/release/bioc/html/trio.html). The correction value for the *trans*-analysis was calculated as the total number of analyzed CpG sites multiplied by the number of all tagSNPs in the whole dataset (pairwise-tagging r^2^<0.9) and subtracted by the correction value for the *cis*-analysis. Significance threshold was set to P<0.05 after correction for multiple testing.

### Expression quantitative trait loci (eQTL) analysis of SNPs identified in the mQTL

To test for associations between SNPs and mRNA expression, an eQTL analysis in the human pancreatic islets including the significant SNPs found to be associated with DNA methylation in the *cis*- or *trans*-mQTL analyses were performed. In the eQTL analyses, significant SNPs identified in the *cis*-mQTL analysis were related to expression of genes in *cis* (≤500 kb between SNP and mRNA transcripts); meanwhile, significant SNPs identified in the *trans*-mQTL were related to expression of all analyzed genes (no distance limit). To test for associations between SNPs and mRNA expression a linear regression assuming an additive genetic model was used. mRNA expression values were used as quantitative trait, SNP genotypes were encoded as 0, 1 or 2 according to the number of minor alleles, and the categorical variable gender as well as the continuous variables age, BMI, HbA1c, islet purity and islet culture days were included as covariates. In the eQTL analysis of significant *cis*-mQTL SNPs, the correction value for multiple testing was calculated by the total sum of tagSNPs within 500 kb to each mRNA transcript in the dataset, where LD pruning of SNPs within a distance of 500 kb from a mRNA transcript was performed by pairwise-tagging with r^2^<0.9. The correction value for multiple testing for the eQTL analysis of significant *trans*-mQTL SNPs was calculated by the number of tagSNPs (LD pruning of included SNPs by pairwise tagging with r^2^<0.9) multiplied by the number of analyzed mRNA transcripts.

### Gene ontology and pathway analyses

Enrichment of gene ontology and/or biological pathways assigned by KEGG was tested among the genes significantly identified in the mQTL and eQTL analyses using Webgestalt (http://bioinfo.vanderbilt.edu/webgestalt, March 2013). The full dataset of analyzed genes in respective mQTL and eQTL analysis was used as background reference. P-values for the KEGG pathway analyses were adjusted for multiple testing using the Benjamini-Hochberg method.

### RNA interference of *Gstt1, Gpx7* and *Snx19* in clonal β-cells

Genes were silenced by siRNA transfection into 832/13 INS-1 β-cells [142] with Dharmafect I (Thermo Scientific, Waltham, MA) according to the manufacturer's instructions. siRNAs (LifeTechnologies, Paisley, UK) used were s151334 (*Gpx7*), s129302 (*Gstt1*), and s164019 (*Snx19*). RNA was isolated 72 h post transfection with the RNeasy Plus mini kit (Qiagen) and converted to cDNA with the RevertAid First Strand cDNA Synthesis kit (Thermo Scientific). Knockdown was quantified by qPCR with the following TaqMan assays (Life Technologies); Rn01416464_m1 (*Gpx7*), Rn00583932_m1 (*Gstt1*), and Rn01524775_m1 (*Snx19*). Assays for *Cyclophilin B* (Rn03302274_m1) and *Hprt1* (Rn01527840_m1) were used as endogenous controls. Quantification was done with the ΔΔCt method.

### Proliferation/apoptosis measurements in clonal β-cells

β-cell number was quantified 72 h post transfection by crystal violet staining as previously described [143], except we used a 0.1% crystal violet solution and read absorbance at 600 nm. The combined activity of caspase-3 and -7 was determined 72 h post transfection with the Apo-One Homogenous Caspase-3/7 assay (Promega, Madison, WI). Lipotoxicity was induced as previously described [144].

### Associations of identified mQTL/eQTL SNPs with islet insulin secretion

To examine if SNPs identified in the mQTL/eQTL analyses were associated with glucose-stimulated insulin secretion in human pancreatic islets cultured *in vitro*, linear regression analyses assuming additive models adjusted for age, sex and BMI were performed. Glucose-stimulated insulin secretion, measured as stimulation index [134], was naturally log transformed before analysis.

### Causal inference test (CIT)

A statistical hypothesis test called CIT [32] was used to distinguish if associations between genotype of SNPs identified in the mQTL analysis and phenotype (gene expression and islet insulin secretion) was potentially mediated by DNA methylation of CpG sites. Each of the genotype (G), methylation (M) and phenotype (Y) relationships were assessed using CIT to classify them as causal (methylation mediated), reactive (methylation consequential) or independent [32]. The statistical test of CIT is based on four mathematical conditions which must be satisfied for the definition of causality: 1) G and Y are associated, 2) G is associated with M after adjusting for Y, 3) M is associated with Y after adjusting for G and 4) G is independent of Y after adjusting for M [32]. A causal call with a hypothesis P-value<0.05 suggests that DNA methylation of a CpG site is a potential mediator between a SNP and phenotype.

### Overlap between identified mQTL/eQTL SNPs and reported diabetes SNPs

The catalog of published genome-wide association studies (GWAS) (www.genome.gov/gwastudies, accessed March 2013) [57] was used to search for SNPs reported to be significantly associated (P<10^−6^) with type 1- and/or type 2 diabetes or diabetes related traits as well as breast cancer, stroke and hypothyroidism used as evaluation references. To gain better reference coverage in the overlap between reported SNPs in the GWAS catalog and identified mQTL/eQTL SNPs in the islets, a SNP annotation and proxy (SNAP) [58] search was performed to identify SNPs in LD with the identified mQTL/eQTL SNPs. The search of LD SNPs was based on pairwise LD calculations of genotype data from the 1000 Genomes project of the CEU population panel, with r^2^ threshold >0.8 and a distance limit of 500 kb from the query SNP. The published diabetes SNPs from the GWAS catalog were then merged with the identified mQTL/eQTL SNPs, together with LD SNPs, to search for overlap between the two datasets.

Publicly available data from the DIAGRAM consortium and MAGIC investigators were also used to look for overlap between identified mQTL-SNPs and SNPs showing associations with diabetes or related traits (P<0.05).

### Associations between DNA methylation and mRNA expression

To test if DNA methylation is directly associated with gene expression in human pancreatic islets, we performed a linear regression between DNA methylation of CpG sites and mRNA transcripts in *cis* (500 kb up- and 100 kb downstream of respective gene), including age, gender, BMI, HbA1c, islet purity, days in culture and batch as covariates.

### Analysis of DNA methylation with Pyrosequencing

Pyrosequencing was used to biologically validate the mQTL data for methylation of two CpG sites annotated to *GPX7* (cg18087326) and *SNX19* (cg08912652). EpiTect Bisulfite Kit (Qiagen) was used for bisulfite conversion of human islet DNA. The PyroMark Assay design Software 2.0 (Qiagen) was used for primer design. PyroSequencing assays (PCR primers and sequencing primer) for the selected CpG sites (Qiagen) can be found in **Table S23**. The PyroMark PCR kit was used for amplification of bisulfite converted DNA. The PyroMark ID 96 and PyroMark Gold Q96 reagents were used for pyrosequencing (Qiagen) according to the manufacturer's instructions. Data were analyzed with the PyroMark Q96 2.5.7 software program.

### Imputation of genotype data

Autosomal genotype data generated with the HumanOmniExpressBeadChip and which passed quality control for the 89 islet donors was imputed to 1000 Genomes phase 1 using Shapeit [145] for phasing and Impute2 for imputation [146]. Imputed data were then filtered based on MAF<0.05 and HWE<0.001.

### Statistical methods

Results are expressed as mean ± sd/sem or Box and Whisker plots. Data were analyzed using linear regression models or Student's t-test. T-statistics are reported from the linear regression analysis, where a t-statistic is defined as the effect size estimate (slope coefficient) divided by its standard error.

## Supporting Information

Figure S1 A correlation heatmap illustrating the overall variability in DNA methylation of all analyzed probes among the 89 donors included in the analyses.
(PNG)

Figure S2 Genomic distribution of CpG sites of significant mQTLs in published data of human adipose tissue from Grundberg et al. 2013. Distribution of CpG sites of significant mQTLs in relation to (A) nearest gene and (B) CpG islands in comparison to all analyzed CpG sites on the Infinium Human Methylation450 BeadChip. (C) Chromosomal distribution of CpG sites of significant mQTLs. mQTL data extracted from publicly available data from Grundberg et al. 2013 [12]. *Significantly different distribution (P<0.05) of CpGs of significant mQTLs from what is expected by chance based on a Chi-squared-test when compared with all analyzed CpG sites on the Infinium HumanMethylation450 BeadChip.
(TIF)

Figure S3 A correlation heatmap illustrating the overall variability in mRNA expression in human pancreatic islets of all analyzed probes among the 89 donors included in the analyses.
(PNG)

Figure S4 Gene Ontology of significant *cis*-mQTLs including genes annotated to the CpG sites showing differential DNA methylation in human pancreatic islets. Analysis performed using Webgestalt (http://bioinfo.vanderbilt.edu/webgestalt, March 2013).
(TIF)

Figure S5 Gene Ontology of genes showing differential expression in the eQTL analysis of *cis*-mQTL-SNPs. Analysis performed using Webgestalt (http://bioinfo.vanderbilt.edu/webgestalt, March 2013).
(TIF)

Figure S6 Gene Ontology of significant *trans*-mQTLs including genes annotated to CpG sites showing differential DNA methylation in human pancreatic islets. Analysis performed using Webgestalt (http://bioinfo.vanderbilt.edu/webgestalt, March 2013).
(TIF)

Figure S7 Gene Ontology of genes showing differential expression in the eQTL analysis of trans-mQTL-SNPs. Analysis performed using Webgestalt (http://bioinfo.vanderbilt.edu/webgestalt, March 2013).
(TIF)

Figure S8 Identification of mQTLs where DNA methylation potentially mediates genetic associations with islet insulin secretion in human pancreatic islets. (A) Depiction of possible relationship models between genotype as a causal factor (G), DNA methylation as a potential mediator (M) and islet insulin secretion as phenotypic outcome (I). Left diagram: The causal or methylation mediated model. Middle diagram: The reactive or methylation-consequential model (reverse causality). Right diagram: The independent model. (B) Illustration of the study approach to identify if DNA methylation of CpG sites potentially mediates the causal association between SNPs and islet insulin secretion. Left: Workflow steps. Middle: Tested relationships between G, M and I in the different steps. Right: Number of identified sites in each step. Bottom: Conditions that must be fulfilled to conclude a mathematical definition of a causal relationship between G, M and I. CIT not corrected for multiple testing and P-value<0.05 considered significant.
(TIF)

Table S1 Islet donor characteristics and glucose-stimulated insulin secretion in human pancreatic islets included in the study.
(PDF)

Table S2 Identified *cis*-mQTLs. (Sheet a) Presents all *cis*-mQTLs showing significant association between SNP genotype and CpG DNA methylation after correction for multiple testing. The maximum distance of 500 kb between SNPs and CpG sites were used to define *cis*-mQTLs. (Sheet b) Annotation of SNPs to significant *cis*-mQTLs. Annotation based on HumanOmniExpress-12v1_J_Gene_Annotation_build37 (Illumina). (Sheet c) Annotation of CpGs to significant *cis*-mQTLs. Annotation based on Infinium HumanMethylation 450 BeadChip (Illumina) [39]. Long stretch enhancers for human pancreatic islets: Based on publicly available data from Parker et al. (2013) [42]. Active enhancer regions in human pancreatic islets: Based on data from Pasquali et al. (2014) [43]. Cross-reactive probes: Maximum number of bases (≥47) matched to cross-reactive target and number of targets as reported by Chen et al.(2013) [33]. Probe SNPs reported by Chen et al. (2013) [33]: SNPs reported by the 1000 Genomes project (release 20110521) that are located within HumanMethylation450 probes, either in sequence of hybridization or at position of single base extension (SBE). Locations of probe-SNPs are presented in relation to MAPINFO of CpG sites, where SBE occurs. Global allele frequencies (AF) and European continental allele frequencies (EUR_AF) of reported probe-SNPs are included in the file.
(XLSX)

Table S3 Identified *trans*-mQTLs. (Sheet a) Presents all *trans*-mQTLs showing significant association between SNP genotype and DNA methylation of CpG sites after correction for multiple testing. All SNP-CpG pairs not located in *cis* were considered as *trans*-mQTLs. (Sheet b) Annotation of SNPs to significant *trans*-mQTLs. Annotation based on HumanOmniExpress-12v1_J_Gene_Annotation_build37 (Illumina). (Sheet c) Annotation of CpGs to significant *trans*-mQTLs. Annotation based on Infinium HumanMethylation 450 BeadChip [39]. Long stretch enhancers for human pancreatic islets: Based on publicly available data from Parker et al. (2013) [42]. Active enhancer regions in human pancreatic islets: Based on data from Pasquali et al. (2014) [43]. Cross-reactive probes: Maximum number of bases (≥47) matched to cross-reactive target and number of targets as reported by Chen et al. (2013) [33]. Probe SNPs reported by Chen et al. (2013) [33]: SNPs reported by the 1000 Genomes project (release 20110521) that are located within HumanMethylation450 probes, either in sequence of hybridization or at position of single base extension (SBE). Locations of probe-SNPs are presented in relation to MAPINFO of CpG sites, where SBE occurs. Global allele frequencies (AF) and European continental allele frequencies (EUR_AF) of reported probe-SNPs are included in the file.
(XLSX)

Table S4 Distribution P-values of CpG sites of significant mQTLs in relation to (A) chromosomes, (B) nearest gene, and (C) CpG islands. *Supporting information to* *Figure 3A, 3C and 3D*.
(PDF)

Table S5 Identified eQTLs of significant *cis*-mQTL-SNPs. (Sheet a) Presents all eQTLs showing significant association between genotype of *cis*-mQTL-SNPs and mRNA expression after correction for multiple testing. SNPs regressed against mRNA expression of mRNA probe sets located in *cis* (≤500 kb). (Sheet b) Annotation of SNPs to significant eQTLs. (Sheet c) Annotation of mRNA probesets to significant eQTLs. Annotation based on HuGene-1_0-st-v1.na32.hg19.transcript (Affymetrix).
(XLSX)

Table S6 Identified eQTLs of significant *trans*-mQTL-SNPs. (Sheet a) Presents all eQTLs showing significant association between genotype of *trans*-mQTL-SNPs and mRNA expression after correction for multiple testing. No distance limit between SNPs and mRNA probesets. (Sheet b) Annotation of SNPs to significant eQTLs. (Sheet c) Annotation of mRNA probesets to significant eQTLs. Annotation based on HuGene-1_0-st-v1.na32.hg19.transcript (Affymetrix).
(XLSX)

Table S7 CIT of significant *cis*-mQTLs/eQTLs identified in human pancreatic islets hypothesizing relationship models between genotypes, DNA methylation and mRNA expression. CIT, causal inference test [32]. Genotype of SNPs identified in the *cis*-mQTL/eQTL analysis are considered as causal factor (G), DNA methylation of CpG sites identified in the *cis*-mQTL analysis as potential mediator (M) and mRNA expression identified in the *cis*-eQTL as phenotypic outcome (E) (*see* *Figure 4A* *for potential relationships between factors*). Called hypothesis models in the CIT analysis: *Causal relationship* (causal P-value<0.05 and reactive P-value>0.05); *Reactive relationship* (causal P-value>0.05 and reactive P-value<0.05); *Independent relationship* (causal P-value>0.05 and reactive P-value>0.05); and No-call (causal P-value<0.05 and reactive P-value<0.05). Highlighted in bold shows causal relationships with FDR<5% (Causal Q-value<0.05).
(XLSX)

Table S8 CIT of significant *trans*-mQTLs/eQTLs identified in human pancreatic islets hypothesizing relationship models between genotypes, DNA methylation and mRNA expression. CIT, causal inference test [32]. Genotype of SNPs identified in the *trans*-mQTL/eQTL analysis are considered as causal factor (G), DNA methylation of CpG sites identified in the *trans*-mQTL analysis as potential mediator (M) and mRNA expression identified in the *trans*-eQTL as phenotypic outcome (E) (*see* *Figure 4A* *for potential relationships between factors*). Called hypothesis models in the CIT analysis: *Causal relationship* (causal P-value<0.05 and reactive P-value>0.05); *Reactive relationship* (causal P-value>0.05 and reactive P-value<0.05); *Independent relationship* (causal P-value>0.05 and reactive P-value>0.05); and No-call (causal P-value<0.05 and reactive P-value<0.05). Highlighted in bold shows causal relationships with FDR<5% (Causal Q-value<0.05).
(XLSX)

Tables S9 KEGG pathways with enrichment of genes showing differential expression between genotype groups in the eQTL analysis of *cis*-mQTL-SNPs. Analysis performed using Webgestalt (http://bioinfo.vanderbilt.edu/webgestalt, March 2013).
(PDF)

Table S10 KEGG pathways with enrichment of genes annotated to CpG sites of significant *trans*-mQTLs in human pancreatic islets. Analysis performed using Webgestalt (http://bioinfo.vanderbilt.edu/webgestalt, March 2013).
(PDF)

Table S11 Associations between significant *cis*-mQTL-SNPs identified in human pancreatic islets and islet insulin secretion.
(XLSX)

Table S12 Associations between significant *trans*-mQTL-SNPs identified in human pancreatic islets and islet insulin secretion.
(XLSX)

Table S13 Identified *cis*-mQTLs where methylation of CpG sites is a potential mediator of genetic association with insulin secretion in human pancreatic islets based on causal inference test *(causal P-value<0.05)*.
(PDF)

Table S14 Overlap between significant *cis*-mQTL-SNPs identified in human pancreatic islets and SNPs reported to associate with type 1 diabetes, type 2 diabetes, glucose traits, insulin traits or proinsulin traits in the GWAS catalog (www.genome.gov/gwastudies, accessed March 2013). (Sheet a) Reported GWAS catalog SNPs or proxy SNPs in linkage (r^2^>0.8) overlapping with significant *cis*-mQTL-SNPs. Proxy search performed by using SNAP (1000 Genomes project, CEU population panel, r^2^>0.8, distance limit 500 kb) [58]. (Sheet b) Extracted information from the GWAS catalog about reported diabetes SNPs.
(XLSX)

Table S15 Overlap between significant *cis*-mQTL-SNPs identified in human pancreatic islets and data from the DIAGRAM consortium or MAGIC investigators. (Sheet a) Association of SNPs with type 2 diabetes reported in DIAGRAM (P<0.05) [59]. Data available at www.diagram-consortium.org. (Sheet b) Association of SNPs with HbA1c [62], (Sheet c) fasting glucose [60], (Sheet d) fasting insulin [60], (Sheet e) HOMA-B [60], (Sheet f) HOMA-IR [60], (Sheet g) fasting proinsulin [63], (Sheet h) BMI adjusted fasting glucose [64], (Sheet i) BMI adjusted fasting insulin [64], and (Sheet j) BMI adjusted 2 h glucose [61] reported in MAGIC (P<0.05). Data downloaded from www.magicinvestigators.org.
(XLSX)

Table S16 Overlap between significant *trans*-mQTL-SNPs identified in human pancreatic islets and data from the DIAGRAM consortium or MAGIC investigators. (Sheet a) Association of SNPs with type 2 diabetes reported in DIAGRAM (P<0.05) [59]. Data available at www.diagram-consortium.org. (Sheet b) Association of SNPs with HbA1c [62], (Sheet c) fasting glucose [60], (Sheet d) fasting insulin [60], (Sheet e) HOMA-B [60], (Sheet f) HOMA-IR [60], (Sheet g) fasting proinsulin [63], (Sheet h) BMI adjusted fasting glucose [64], (Sheet i) BMI adjusted fasting insulin [64], and (Sheet j) BMI adjusted 2 h glucose [61] reported in MAGIC (P<0.05). Data downloaded from www.magicinvestigators.org.
(XLSX)

Table S17 Associations between DNA methylation and mRNA expression in human pancreatic islets. (Sheet a) All significant combinations of CpG sites and mRNA expression probe-sets showing associations between DNA methylation mRNA expressions after correction for multiple testing using false discovery rate <5%. (Sheet b) Merged mQTL/eQTL data where CpG sites and mRNA expression probe-sets where both were significantly affected by the same SNP. (Sheet c) Overlap between mQTL/eQTL data and direct association between DNA methylation and mRNA levels.
(XLSX)

Table S18 Islet donor characteristics and glucose-stimulated insulin secretion in human pancreatic islets included in the validation cohort.
(PDF)

Table S19 Overlap between significant CpG sites in our *cis*-mQTL study in human pancreatic islets and previously published *cis*-mQTL studies in other human tissues. Previously published human mQTL studies in the overlap analysis includes: Zhang et al. 2010 [8]; Gibbs et al. 2010 [9]; Gutierrez-Arceleus et al. 2013 [13]; Grundberg et al. 2013 [12]; and Wagner et al. 2014 [14].
(PDF)

Table S20 KEGG pathways with enrichment of genes annotated to CpG sites of significant *cis*-mQTLs only identified in human pancreatic islets (i.e. the pathway analysis includes CpG sites in significant *cis*-mQTLs annotated to unique genes in our islet mQTL analysis that cannot be replicated in any previously published human mQTL study [8], [9], [12]–[14]. Analysis performed using Webgestalt (http://bioinfo.vanderbilt.edu/webgestalt, June 2013).
(PDF)

Table S21 Identified *cis*-mQTLs of imputed genotype data. Presents all *cis*-mQTLs showing significant association between SNP genotype including imputed genotype data and CpG DNA methylation after correction for multiple testing. Imputed autosomal genotype data generated with the HumanOmniExpressBeadChip for islet donors to the 1000 Genomes phase 1. The maximum distance of 500 kb between SNPs and CpG sites were used to define *cis*-mQTLs. Annotation of SNPs to significant *cis*-mQTLs based on genome build 37. Annotation of CpGs based on genome build 37 and Infinium HumanMethylation 450 BeadChip (Illumina) [39]. Note: Data file is large (>90 MB).
(XLSX)

Table S22 Identified *trans*-mQTLs of imputed genotype data. Presents all *trans*-mQTLs showing significant association between SNP genotype including imputed genotype data and CpG DNA methylation after correction for multiple testing. Imputed autosomal genotype data generated with the HumanOmniExpressBeadChip for islet donors to the 1000 Genomes phase 1. All SNP-CpG pairs not located in *cis* were considered as *trans*-mQTLs. Annotation of SNPs to significant *cis*-mQTLs based on genome build 37. Annotation of CpGs based on genome build 37 and Infinium HumanMethylation 450 BeadChip (Illumina) [39].
(XLSX)

Table S23 DNA sequences for pyrosequencing forward, reverse and sequencing primers.
(XLSX)
