# Correction: Genome-Wide Associations between Genetic and Epigenetic Variation Influence mRNA Expression and Insulin Secretion in Human Pancreatic Islets

**DOI:** 10.1371/journal.pgen.1004886

**Published:** 2014-12-12

**Authors:** The PLOS Genetics Staff

Due to errors in production, Figure 2 and Figure 4 are incorrect. The correct versions are provided here.

**Figure 2:**
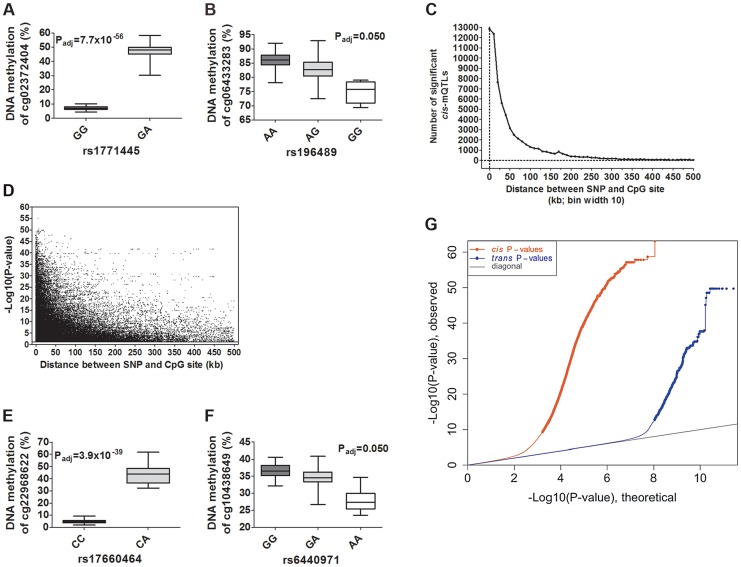
Depiction and distance analysis of associations between genotype and DNA methylation of significant mQTLs in human pancreatic islets. Depiction of (**A**) the most significant *cis*-mQTL; rs1771445 vs. cg02372404, and (**B**) the least significant *cis*-mQTL; rs196489 vs. cg06433283, among all identified *cis*-mQTLs in human pancreatic islets. Data is presented as Box and Whisker plots with P-values adjusted for multiple testing. (**C**) Distance analysis between SNPs and CpG sites of significant *cis*-mQTLs plotted as the number of identified mQTLs within each distance bin. Distance summary: minimum  =  0 kb, 10%ile  =  1.88 kb, 25%ile  =  7.62 kb, 50%ile  =  26.31 kb, 75%ile  =  74.76 kb, 90%ile  =  164.5 kb, maximum  =  499.6 kb. (**D**) The strength of associations plotted against the distance between SNPs and CpG sites of significant *cis*-mQTLs after correction for multiple testing. Depiction of (**E**) the most significant *trans*-mQTL; rs17660464 vs. cg22968622, and (**F**) the least significant *trans*-mQTL; rs6440971 vs. cg10438649, among all identified *trans*-mQTLs in human pancreatic islets. Data is presented as Box and Whisker plots with P-values adjusted for multiple testing. (**G**) Quantile-Quantile plots (Q-Q plots) of –log10 (P-values) illustrating the distribution of P-values for all analyzed SNP-CpG pairs in the *cis*- (red dots) and *trans*- (blue dots) mQTL analysis in relation to a theoretical null distribution (grey diagonal line). Bold dots indicate significant mQTLs identified in the *cis*- (red dots) and *trans*-(blue dots) mQTL analysis after correction for multiple testing.

**Figure 4:**
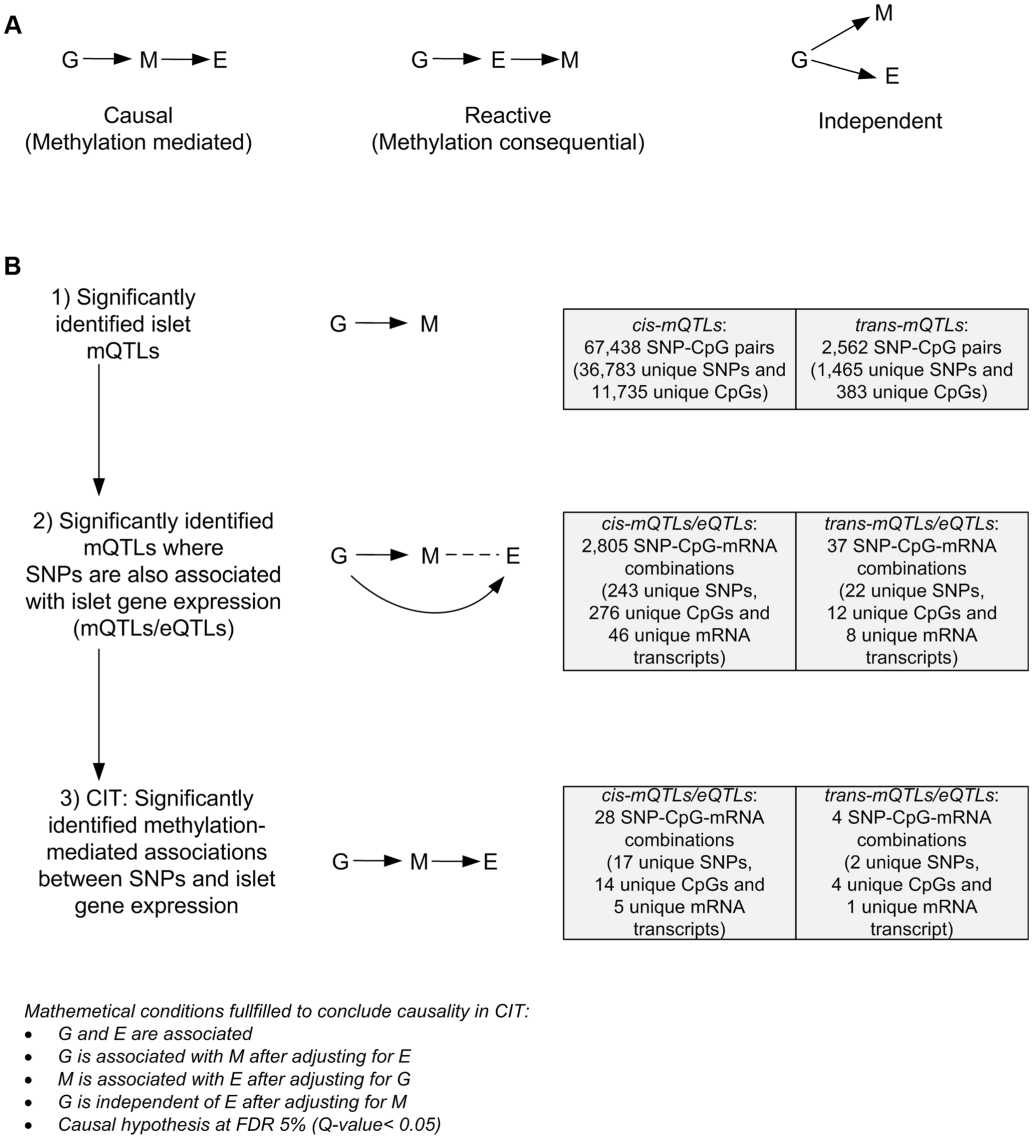
CIT analysis identifies mQTLs where DNA methylation potentially mediates genetic associations with mRNA expression in human pancreatic islets. (**A**) Depiction of possible relationship models between genotype as a causal factor (G), DNA methylation as a potential mediator (M) and islet mRNA expression as a phenotypic outcome (E). Left diagram: The causal or methylation mediated model. Middle diagram: The reactive or methylation-consequential model (reverse causality). Right diagram: The independent model. (**B**) Illustration of the study approach to identify if DNA methylation of CpG sites potentially mediates the causal association between SNPs and islet mRNA expression. Left: Workflow steps. Middle: Tested relationships between G, M and E in the different steps. Right: Number of identified sites in each step. Bottom: Conditions that must be fulfilled to conclude a mathematical definition of a causal relationship between G, M and E. Significantly called as causal at 5% FDR (causal hypothesis Q<0.05).
