# Supplementary figures and images for: Genome-Wide Associations between Genetic and Epigenetic Variation Influence mRNA Expression and Insulin Secretion in Human Pancreatic Islets
Source: PLoS Genet. 2014 Nov 6;10(11):e1004735. doi: 10.1371/journal.pgen.1004735 (PMC4222689; doi:10.1371/journal.pgen.1004735)

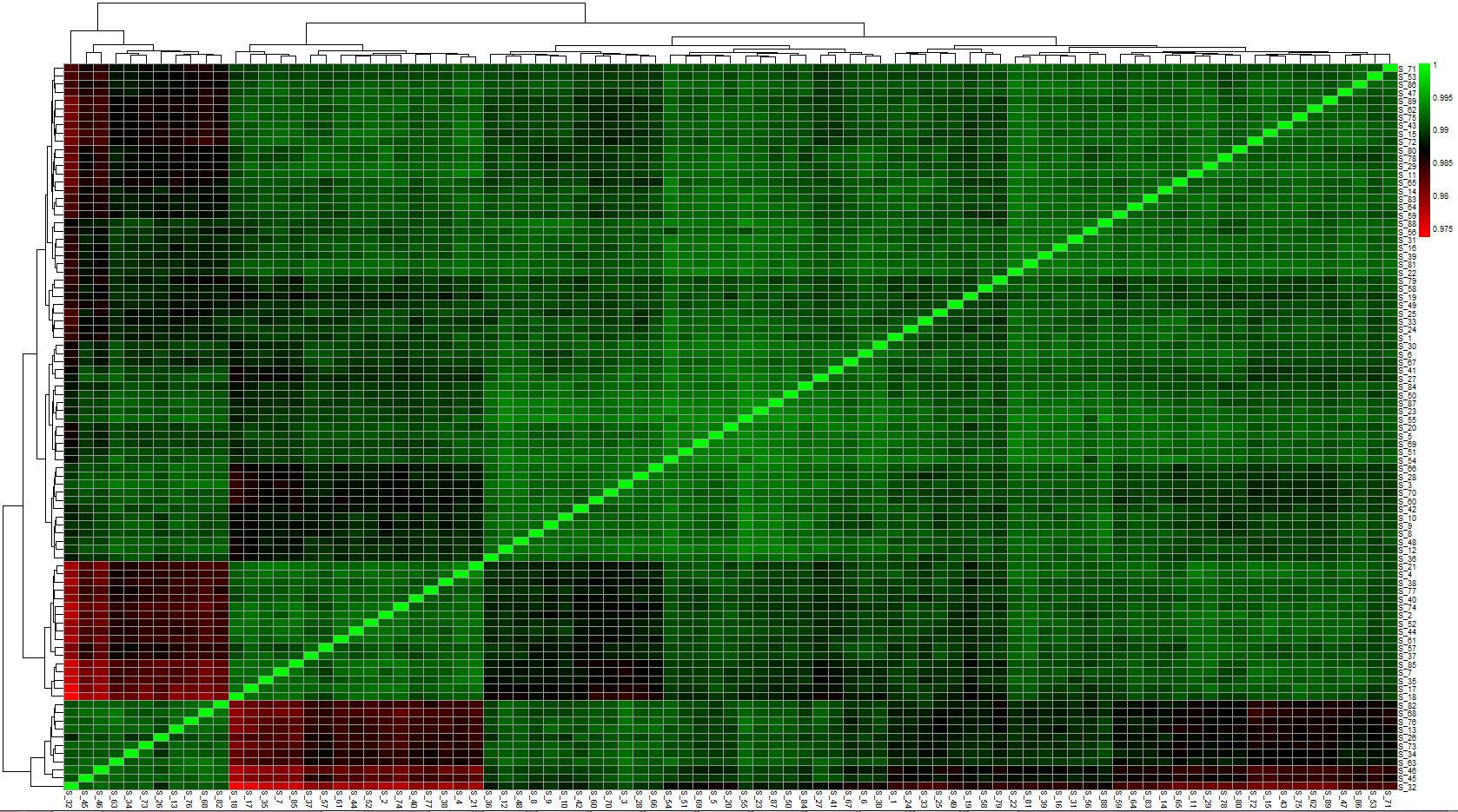

Supplement: Figure S1 — A correlation heatmap illustrating the overall variability in DNA methylation of all analyzed probes among the 89 donors included in the analyses. (PNG) [file pgen.1004735.s001.png]

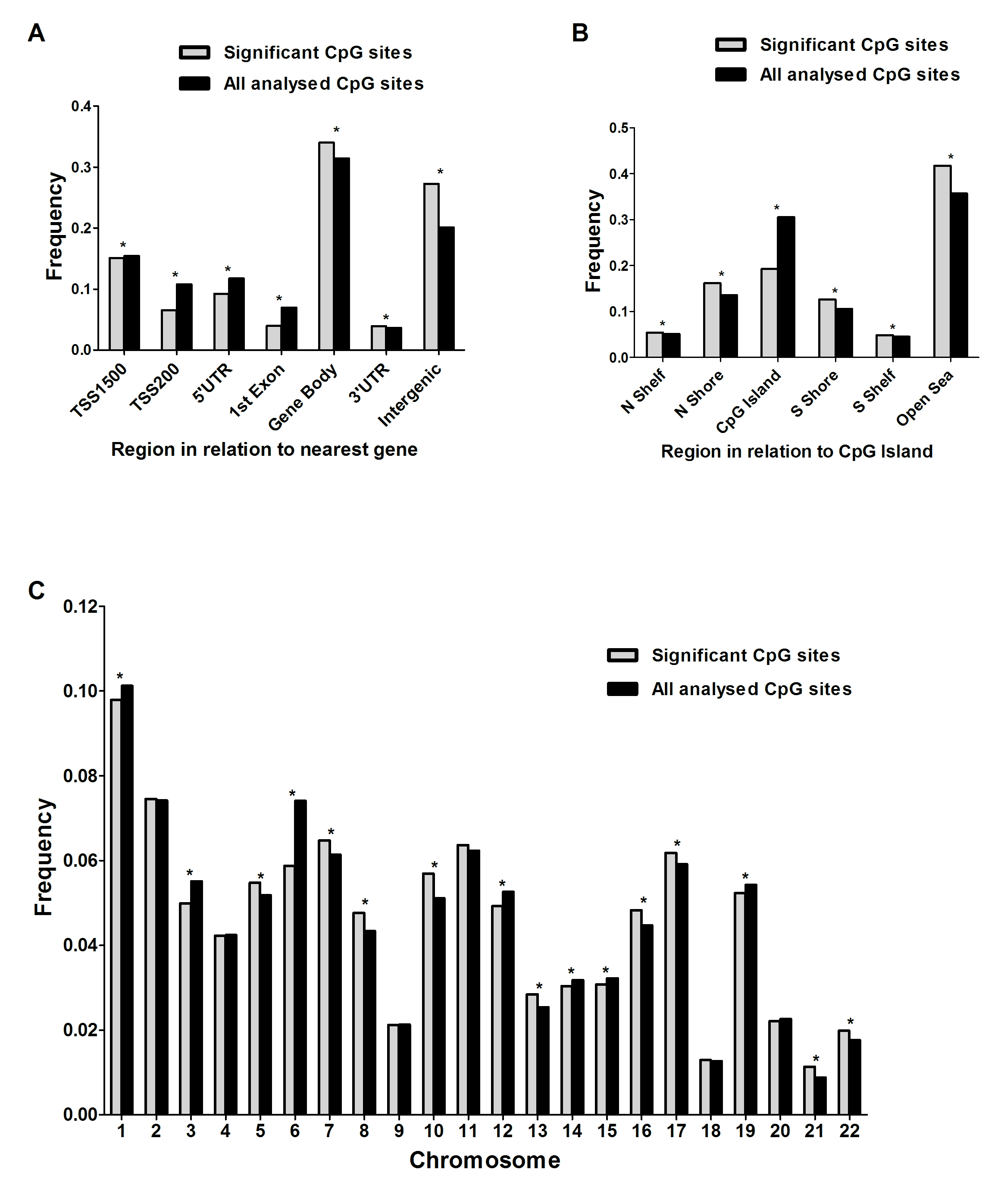

Supplement: Figure S2 — Genomic distribution of CpG sites of significant mQTLs in published data of human adipose tissue from Grundberg et al. 2013. Distribution of CpG sites of significant mQTLs in relation to (A) nearest gene and (B) CpG islands in comparison to all analyzed CpG sites on the Infinium Human Methylation450 BeadChip. (C) Chromosomal distribution of CpG sites of significant mQTLs. mQTL data extracted from publicly available data from Grundberg et al. 2013 [12]. *Significantly different distribution (P<0.05) of CpGs of significant mQTLs from what is expected by chance based on a Chi-squared-test when compared with all analyzed CpG sites on the Infinium HumanMethylation450 BeadChip. (TIF) [file pgen.1004735.s002.tif]

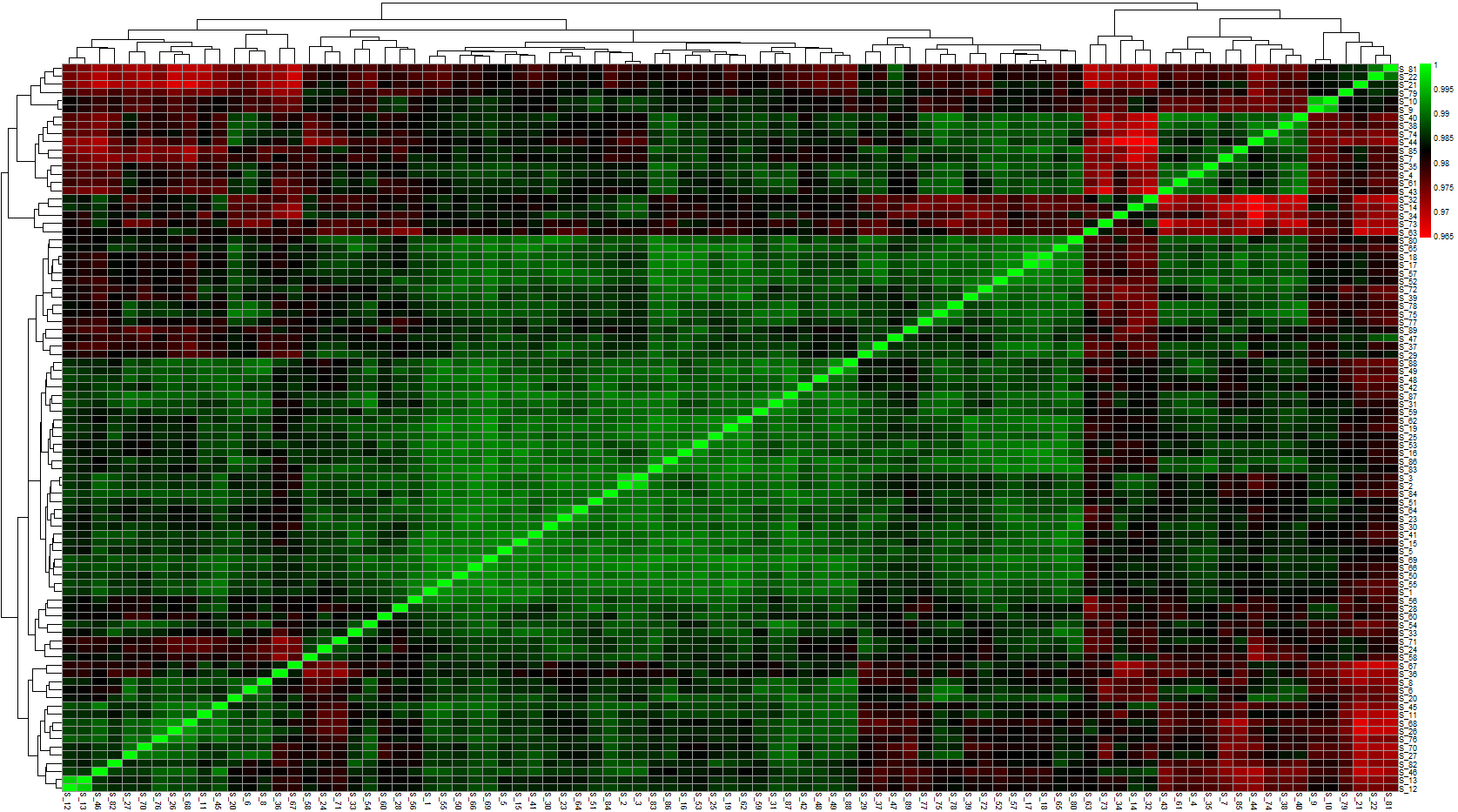

Supplement: Figure S3 — A correlation heatmap illustrating the overall variability in mRNA expression in human pancreatic islets of all analyzed probes among the 89 donors included in the analyses. (PNG) [file pgen.1004735.s003.png]

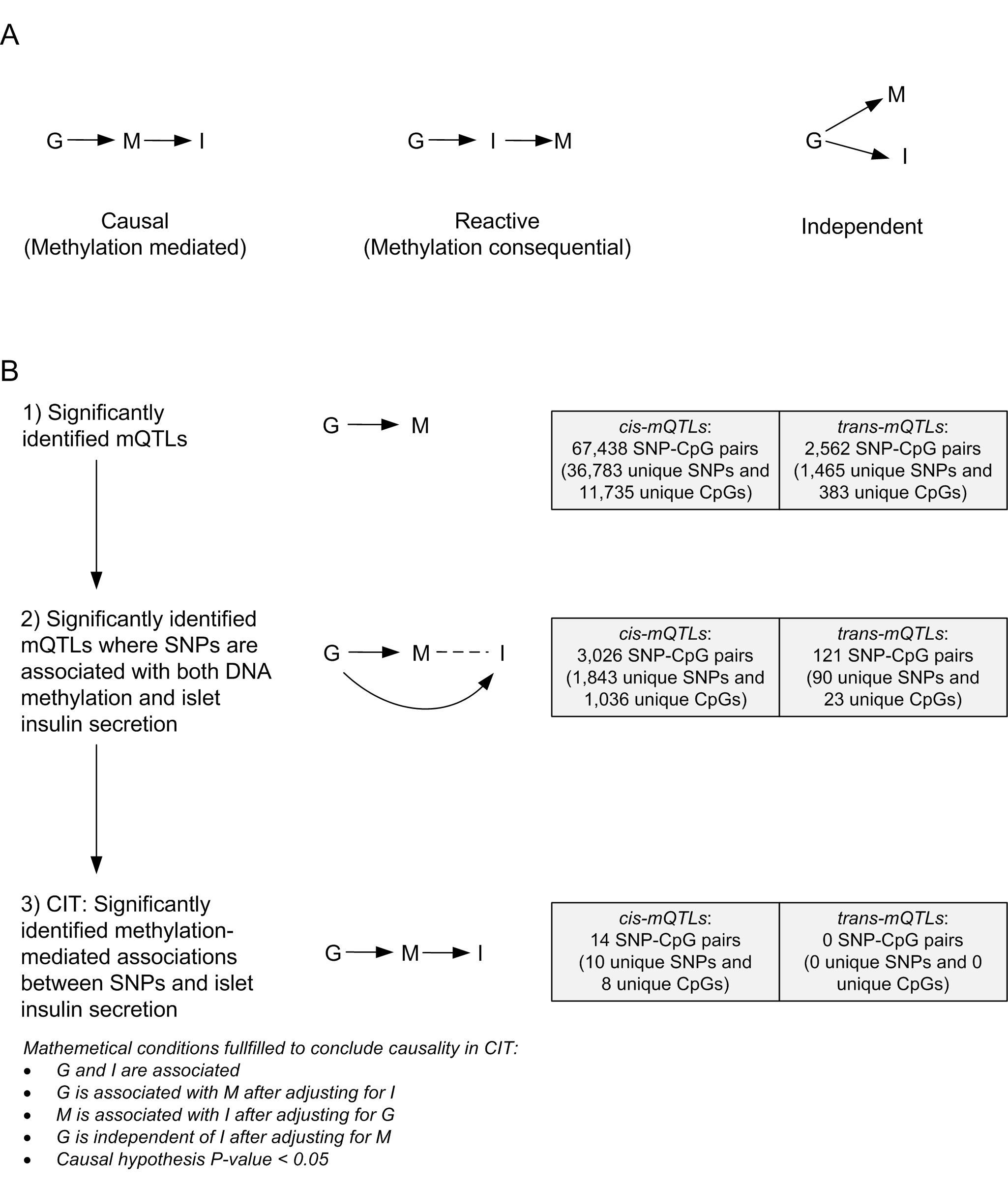

Supplement: Figure S8 — Identification of mQTLs where DNA methylation potentially mediates genetic associations with islet insulin secretion in human pancreatic islets. (A) Depiction of possible relationship models between genotype as a causal factor (G), DNA methylation as a potential mediator (M) and islet insulin secretion as phenotypic outcome (I). Left diagram: The causal or methylation mediated model. Middle diagram: The reactive or methylation-consequential model (reverse causality). Right diagram: The independent model. (B) Illustration of the study approach to identify if DNA methylation of CpG sites potentially mediates the causal association between SNPs and islet insulin secretion. Left: Workflow steps. Middle: Tested relationships between G, M and I in the different steps. Right: Number of identified sites in each step. Bottom: Conditions that must be fulfilled to conclude a mathematical definition of a causal relationship between G, M and I. CIT not corrected for multiple testing and P-value<0.05 considered significant. (TIF) [file pgen.1004735.s008.tif]
