# Supplementary material for: Genome-Wide Associations between Genetic and Epigenetic Variation Influence mRNA Expression and Insulin Secretion in Human Pancreatic Islets
Source: PLoS Genet. 2014 Nov 6;10(11):e1004735. doi: 10.1371/journal.pgen.1004735 (PMC4222689; doi:10.1371/journal.pgen.1004735)
Supplement: Table S1 — Islet donor characteristics and glucose-stimulated insulin secretion in human pancreatic islets included in the study. (PDF) [file pgen.1004735.s009.pdf]

Table S1 Islet donor characteristics and glucose-stimulated insulin secretion in human pancreatic islets included in the study

n (male/female)	89 (55/34)
Age (years)	57.0 ± 10.5
BMI (kg/m^2)	25.8 ± 3.4
HbA1c (%)	5.7 ± 0.7
HbA1c (mmol/mol)	49 ± 7
Glucose-stimulated insulin secretion in islets – Stimulation Index (SI)	6.9 ± 5.6

Data are expressed as mean ± SD

HbA_{1c} was measured with the Mono-S method
