# Supplementary material for: Genome-Wide Associations between Genetic and Epigenetic Variation Influence mRNA Expression and Insulin Secretion in Human Pancreatic Islets
Source: PLoS Genet. 2014 Nov 6;10(11):e1004735. doi: 10.1371/journal.pgen.1004735 (PMC4222689; doi:10.1371/journal.pgen.1004735)
Supplement: Table S4 — Distribution P-values of CpG sites of significant mQTLs in relation to (A) chromosomes, (B) nearest gene, and (C) CpG islands. Supporting information to Figure 3A, 3C and 3D . (PDF) [file pgen.1004735.s012.pdf]

Table S4A Distribution of P-values of CpG sites of significant mQTLs in relation to chromosomes (*Supporting information to Figure 3A*)

Chromosome	P-values *cis*-mQTL[1]	P-values *trans*-mQTL[2]
1	0.0050	0.0038
2	0.0122	0.5480
3	6.2655E-06	0.0810
4	0.5147	0.7098
5	0.9818	0.2831
6	1.5620E-70	1.4977E-07
7	0.0337	0.7167
8	0.0082	0.6356
9	0.0772	0.0333
10	0.1212	0.8726
11	0.2817	0.4315
12	0.0025	0.5137
13	0.1537	0.7685
14	0.0052	0.0171
15	0.0179	0.1004
16	0.0225	0.4967
17	0.0111	0.0136
18	0.0932	0.4089
19	0.0288	0.8705
20	0.0018	0.4046
21	0.0003	0.1878
22	0.7949	0.7269

P-values of difference in the distribution of CpGs of significant [1]*cis*- or [2]*trans*-mQTLs from what expected by chance based on Chi-squared-test when compared with all analyzed CpG sites on the Infinium HumanMethylation450 BeadChip.

Table S4B Distribution of P-values of CpG sites of significant mQTLs in relation to nearest gene (*Supporting information to Figure 3C*)

Gene group	P-values *cis*-mQTL[1]	P-values *trans*-mQTL[2]
TSS1500	0.0217	0.0028
TSS200	2.4940E-40	0.6186
5'UTR	2.7889E-33	0.3254
1stExon	3.1591E-50	0.0056
Body	9.2040E-06	0.0733
3'UTR	0.0116	0.2578
Intergenic	9.7261E-160	1.4778E-51

P-values of difference in the distribution of CpGs of significant [1]*cis*- or [2]*trans*-mQTLs from what expected by chance based on Chi-squared-test when compared with all analyzed CpG sites on the Infinium HumanMethylation450 BeadChip.

Table S4C Distribution of P-values of CpG sites of significant mQTLs in relation to CpG Islands (*Supporting information to Figure 3D*)

CpG island group	Obs freq[1]	Exp freq[2]
N Shelf	0.3555	0.0521
N Shore	0.0070	0.0016
Island	8.7333E-114	0.0013
S Shore	1.3194E-05	0.2423
S Shelf	3.2497E-04	0.6948
Open Sea	2.7420E-52	0.3811

P-values of difference in the distribution of CpGs of significant [1]*cis*- or [2]*trans*-mQTLs from what expected by chance based on Chi-squared-test when compared with all analyzed CpG sites on the Infinium HumanMethylation450 BeadChip.
