# Supplementary material for: Genome-Wide Associations between Genetic and Epigenetic Variation Influence mRNA Expression and Insulin Secretion in Human Pancreatic Islets
Source: PLoS Genet. 2014 Nov 6;10(11):e1004735. doi: 10.1371/journal.pgen.1004735 (PMC4222689; doi:10.1371/journal.pgen.1004735)
Supplement: Tables S9 — KEGG pathways with enrichment of genes showing differential expression between genotype groups in the eQTL analysis of cis-mQTL-SNPs. Analysis performed using Webgestalt (http://bioinfo.vanderbilt.edu/webgestalt, March 2013). (PDF) [file pgen.1004735.s017.pdf]

Table S9 KEGG pathways with enrichment of genes showing differential expression between genotype groups in the eQTL analysis of *cis*-mQTL-SNPs

**Pathway (total number of genes in pathway)**	**Observed number of genes**	**Expected number of genes**	**Ratio of enrichment**	**Raw *P*-value**	**Adjusted *P*-value**	**Observed genes**
Glutathione metabolism (45)	3	0.08	35.56	8.09×10^{-5}	0.0003	*GSTT1, GSTM3, GPX7*
Drug metabolism - cytochrome P450 (53)	2	0.10	20.13	0.0044	0.0059	*GSTT1, GSTM3*
Metabolism of xenobiotics by cytochrome P450 (53)	2	0.10	20.13	0.0044	0.0059	*GSTT1, GSTM3*

P-values have been adjusted for multiple testing using Benjamini-Hochberg
