# Supplementary material for: Genome-Wide Associations between Genetic and Epigenetic Variation Influence mRNA Expression and Insulin Secretion in Human Pancreatic Islets
Source: PLoS Genet. 2014 Nov 6;10(11):e1004735. doi: 10.1371/journal.pgen.1004735 (PMC4222689; doi:10.1371/journal.pgen.1004735)
Supplement: Table S10 — KEGG pathways with enrichment of genes annotated to CpG sites of significant trans-mQTLs in human pancreatic islets. Analysis performed using Webgestalt (http://bioinfo.vanderbilt.edu/webgestalt, March 2013). (PDF) [file pgen.1004735.s018.pdf]

Table S10 KEGG pathways with enrichment of genes annotated to CpG sites of significant *trans*-mQTLs in human pancreatic islets

**Pathway (total number of genes in pathway)**	**Observed number of genes**	**Expected number of genes**	**Ratio of enrichment**	**Raw *P*-value**	**Adjusted *P*-value**	**Observed genes**
Type 1 Diabetes (41)	6	0.51	11.79	1.07×10^{-5}	0.0006	*PTPRN2, HLA-DRB1, HLA-B, HLA-C, HSPD1, HLA-DRB5*
Autoimmune thyroid disease (41)	4	0.51	7.86	0.0016	0.018	*HLA-DRB1, HLA-B, HLA-C, HLA-DRB5*
Antigen processing and presentation (68)	5	0.84	5.93	0.0016	0.018	*HLA-DRB1, HLA-B, TAPBP, HLA-C, HLA-DRB5*
Allograft rejection (34)	4	0.42	9.48	0.0008	0.018	*HLA-DRB1, HLA-B, HLA-C, HLA-DRB5*
Graft versus host disease (37)	4	0.46	8.71	0.0011	0.018	*HLA-DRB1, HLA-B, HLA-C, HLA-DRB5*
Systemic lupus erythematosus (124)	6	1.54	3.90	0.0045	0.042	*HLA-DRB1, HIST1H2AM, HIST1H2BI, HLA-DRB5, HIST1H4D, HIST1H2BO*

P-values have been adjusted for multiple testing using Benjamini-Hochberg
