# Supplementary material for: Genome-Wide Associations between Genetic and Epigenetic Variation Influence mRNA Expression and Insulin Secretion in Human Pancreatic Islets
Source: PLoS Genet. 2014 Nov 6;10(11):e1004735. doi: 10.1371/journal.pgen.1004735 (PMC4222689; doi:10.1371/journal.pgen.1004735)
Supplement: Table S13 — Identified cis-mQTLs where methylation of CpG sites is a potential mediator of genetic association with insulin secretion in human pancreatic islets based on causal inference test (causal P-value<0.05). (PDF) [file pgen.1004735.s021.pdf]

Table S13 Identified *cis*-mQTLs where methylation of CpG sites is potential mediators of genetic association with insulin secretion in human pancreatic islets

CpG					SNP			G vs M		G vs I		CIT
**CpG Id**	**Gene**	**Gene Region**	**Chr**	**Pos**	**Id**	**Chr**	**Pos**	**T-Stat**	**P-Value**	**T-Stat**	**P-Value**	**P-Value**
cg23231268	-	Intergenic	3	46792462	rs6763438	3	46778988	-7.26	0.022	-0.38	0.010	0.011
cg23231268	-	Intergenic	3	46792462	rs7614762	3	46790397	-7.26	0.022	-0.38	0.010	0.011
cg23231268	-	Intergenic	3	46792462	rs6810260	3	46783432	-7.56	0.0056	-0.41	0.006	0.016
cg07207484	*PTPRN2*	Gene Body	7	158316262	rs7810884	7	158276926	-8.47	8.99E-05	0.33	0.025	0.017
cg08381504	-	Intergenic	3	46792357	rs6763438	3	46778988	-7.46	0.0088	-0.38	0.010	0.022
cg08381504	-	Intergenic	3	46792357	rs7614762	3	46790397	-7.46	0.0088	-0.38	0.010	0.022
cg08381504	-	Intergenic	3	46792357	rs6810260	3	46783432	-8.27	0.00023	-0.41	0.006	0.032
cg05124692	*PADI4*	TSS1500	1	17633195	rs2477142	1	17629004	-10.36	1.72E-08	-0.41	0.000	0.034
cg02884280	*LOC100130581*	TSS200	17	41466285	rs6503721	17	41127448	7.61	0.0044	0.28	0.025	0.039
cg02884280	*LOC100130581*	TSS200	17	41466285	rs455055	17	41167957	8.32	0.00018	0.33	0.011	0.042
cg27396498	*CES4*	TSS200	16	55794478	rs8192946	16	55848218	-8.46	9.50E-05	-0.35	0.020	0.042
cg27396498	*CES4*	TSS200	16	55794478	rs1968753	16	55845351	-8.62	4.56E-05	-0.36	0.013	0.043
cg22309949	-	Intergenic	3	46792192	rs6810260	3	46783432	-7.97	0.00088	-0.41	0.006	0.046
cg10769313	-	Intergenic	19	15871084	rs10426753	19	15886084	7.35	0.015	-0.32	0.011	0.048

Causal Inference Test (CIT) was used to test if associations between SNPs identified in the *cis*-mQTL analysis and islets insulin secretion was mediated by DNA methylation of CpG sites.
G vs M: Adjusted associations between genotype (G) and DNA methylation (M) – *cis*-mQTL analysis. P-values corrected for multiple testing based on number of independent tests performed.
G vs I: Adjusted associations between genotype (G) and human islet insulin secretion measured as stimulation index (I).
CIT: Causal hypothesis P-value < 0.05 showing that genotype (G) and islet insulin secretion (I) relationship is considered mediated through DNA methylation (M).
