# Supplementary material for: Genome-Wide Associations between Genetic and Epigenetic Variation Influence mRNA Expression and Insulin Secretion in Human Pancreatic Islets
Source: PLoS Genet. 2014 Nov 6;10(11):e1004735. doi: 10.1371/journal.pgen.1004735 (PMC4222689; doi:10.1371/journal.pgen.1004735)
Supplement: Table S18 — Islet donor characteristics and glucose-stimulated insulin secretion in human pancreatic islets included in the validation cohort. (PDF) [file pgen.1004735.s026.pdf]

Table S18 Islet donor characteristics and glucose-stimulated insulin secretion in human pancreatic islets included in the validation cohort

n (male/female)	37 (19/18)
Age (years)	59.3 ± 10.3
BMI (kg/m^2)	28.0 ± 4.2
HbA1c (%)	6.3 ± 0.86
HbA1c (mmol/mol)	55 ± 9
Glucose-stimulated insulin secretion in islets – Stimulation Index (SI)	6.5 ± 5.3

Data are expressed as mean ± SD

HbA_{1c} was measured with the Mono-S method
