# Supplementary material for: Genome-Wide Associations between Genetic and Epigenetic Variation Influence mRNA Expression and Insulin Secretion in Human Pancreatic Islets
Source: PLoS Genet. 2014 Nov 6;10(11):e1004735. doi: 10.1371/journal.pgen.1004735 (PMC4222689; doi:10.1371/journal.pgen.1004735)
Supplement: Table S19 — Overlap between significant CpG sites in our cis-mQTL study in human pancreatic islets and previously published cis-mQTL studies in other human tissues. Previously published human mQTL studies in the overlap analysis includes: Zhang et al. 2010 [8]; Gibbs et al. 2010 [9]; Gutierrez-Arceleus et al. 2013 [13]; Grundberg et al. 2013 [12]; and Wagner et al. 2014 [14]. (PDF) [file pgen.1004735.s027.pdf]

Table S19 Overlap between significant CpG sites in our *cis*-mQTL study in human pancreatic islets and previously published *cis*-mQTL studies in other human tissues

Study	Methylation array	Human samples	*cis* distance	Significance threshold	Nr of significant CpG sites	Nr of CpG sites replicated in our study
Olsson et al. 2014	Illumina 450k	89 pancreatic islets	500kb	Correction for # of independent tests corresponding to p-value $\leq 4.9 \cdot 10^{-10}$	11,735[a]	-
Zhang et al. 2010	Illumina 27k	153 brain cerebellum	1 Mb	Region-wide permuted p-value ≤0.05 (and phenotype-wide corrected p-value ≤0.05)	2,046 (736)	143 (102)
Gibbs et al. 2010	Illumina 27k	108 brain cerebellum	1 Mb	Genome-wide permuted empirical p-value ≤0.02697	444	96
-||-	-||-	133 brain frontal cortex	1 Mb	Genome-wide permuted empirical p-value ≤0.02597	420	111
-||-	-||-	125 brain pons	1 Mb	Genome-wide permuted empirical p-value ≤0.02697	359	109
-||-	-||-	127 brain temporal cortex	1 Mb	Genome-wide permuted empirical p-value ≤0.02697	547	126
Gutierrez-Arceleus et al. 2013	Illumina 450k	107 fibroblast	5 kb	FDR 10% correspondning to p-value $\leq 4.4 \cdot 10^{-4}$	14,189	2,189
	-||-	111 lymphoblastoid cell	-||-	FDR 10% correspondning to p-value $\leq 7.9 \cdot 10^{-4}$	22,411	2,467
	-||-	66 T-cell	-||-	FDR 10% correspondning to p-value $\leq 1.3 \cdot 10^{-3}$	32,318	2,780
Grundberg et al. 2013	Illumina 450k	603 adipose tissue	100 kb	FDR 1% corresponding to p-value $< 8.6 \cdot 10^{-4}$	98,085	3,888

Wagner et al. 2014[b]	Illumina 450k	62 primary fibroblast	250 kb	FDR 5% corresponding to p-value $\leq 6 \cdot 10^{-6}$	1,676	651

[a]339 of the loci were included on the Illumina 27k methylation array.
[b]Only probes showing variance across samples in the top 25% were included in the mQTL analysis.
FDR=false discovery rate.
Note: Different inclusion criteria of probes, different *cis* windows and different thresholds for calling significant mQTL hits are not considered in the replication.
