# Supplementary material for: Genome-Wide Associations between Genetic and Epigenetic Variation Influence mRNA Expression and Insulin Secretion in Human Pancreatic Islets
Source: PLoS Genet. 2014 Nov 6;10(11):e1004735. doi: 10.1371/journal.pgen.1004735 (PMC4222689; doi:10.1371/journal.pgen.1004735)
Supplement: Table S20 — KEGG pathways with enrichment of genes annotated to CpG sites of significant cis-mQTLs only identified in human pancreatic islets (i.e. the pathway analysis includes CpG sites in significant cis-mQTLs annotated to unique genes in our islet mQTL analysis that cannot be replicated in any previously published human mQTL study [8], [9], [12]–[14]. Analysis performed using Webgestalt (http://bioinfo.vanderbilt.edu/webgestalt, June 2013). (PDF) [file pgen.1004735.s028.pdf]

Table S20 KEGG pathways with enrichment of genes annotated to CpG sites of significant *cis*-mQTLs only identified in human pancreatic islets.

**Pathway (total number of genes in pathway)**	**Observed number of genes**	**Expected number of genes**	**Ratio of enrichment**	**Raw *P*-value**	**Adjusted *P*-value**	**Observed genes**
Type I diabetes mellitus	23	6.59	3.49	$5.23x10^{-9}$	$5.44x10^{-7}$	*HLA-DRA, HLA-DQA2, HLA-DQA1, CD86, HLA-E, HLA-F, CD80, IL1A, HLA-DPB1, HLA-A, INS, HLA-DRB1, HLA-DMA, HLA-DPA1, HLA-B, ICA1, HLA-DQB1, PTPRN2, HLA-G, HLA-C, HLA-DOA, HLA-DOB, HLA-DRB5*
Autoimmune thyroid disease	23	6.59	3.49	$5.23x10^{-9}$	$5.44x10^{-7}$	*HLA-DRA, HLA-DQA2, CTLA4, HLA-DQA1, CD86, HLA-E, HLA-F, CD80 ,TPO, HLA-DPB1, HLA-A, HLA-DRB1, HLA-DMA, HLA-DPA1, HLA-B, HLA-DQB1 ,HLA-G, TG, HLA-C, HLA-DOA, HLA-DOB, HLA-DRB5, IFNA4*
Allograft rejection	19	5.46	3.48	1.24x10-7	6.45x10-6	*HLA-DRA, HLA-DQA2, HLA-DQA1, CD86, HLA-E, HLA-F, CD80, HLA-DPB1, HLA-A, HLA-DMA, HLA-DPA1, HLA-DRB1, HLA-B, HLA-DQB1, HLA-G, HLA-C, HLA-DOA, HLA-DOB, HLA-DRB5*
Graft-versus-host disease	20	5.95	3.36	1.21x10-7	6.45x10-6	*HLA-DRA, HLA-DQA2, HLA-DQA1, CD86, HLA-E, HLA-F, CD80, IL1A, HLA-DPB1, HLA-A, HLA-DMA, HLA-DPA1, HLA-DRB1, HLA-B, HLA-DQB1, HLA-G, HLA-C, HLA-DOA, HLA-DOB, HLA-DRB5*
Viral myocarditis	26	10.61	2.45	4.50x10-6	0.0002	*HLA-DRA, HLA-DQA2, CASP3, HLA-DQA1, CD86, HLA-E, HLA-F, SGCD, CD80, CAV1, LAMA2, HLA-DPB1, HLA-A, MYH11, HLA-DRB1, HLA-DMA, HLA-DPA1, ITGB2, HLA-B, FYN, HLA-DQB1, HLA-G, HLA-C, HLA-DOA, HLA-DRB5, HLA-DOB*
Antigen processing and presentation	26	10.93	2.38	8.55x10-6	0.0003	*HLA-DRA, HLA-DQA2, HLA-DQA1, HLA-E, HLA-F, HSP90AB1, TAP1, HLA-DPB1, HLA-A, HSPA1B, HLA-DRB1, HLA-DMA, HLA-DPA1, HSPA1L, HLA-B, TAPBP, NFYA, HLA-DQB1, HLA-G, TAP2, HLA-C, HSP90AA1, HLA-DOA, HLA-DRB5, KLRC2, HLA-DOB*

Cell adhesion molecules (CAMs)	38	20.09	1.89	4.45x10-5	0.0012	*HLA-DRA, CDH4, CLDN15, CD86, HLA-E, HLA-F, CD276, SELL, CNTNAP2, NRXN1 ,CLDN14, HLA-DPB1, HLA-A, HLA-DRB1, HLA-DPA1, CDH15, CDH1, HLA-DQB1, CLDN4, HLA-C, ITGA9, NCAM1, CLDN18, HLA-DQA2, CTLA4, HLA-DQA1, ICAM3, CD80, NEGR1, ITGB2, HLA-DMA, PTPRF, HLA-B, HLA-G, NFASC, HLA-DOA, HLA-DOB, HLA-DRB5*
Phagosome	42	22.98	1.83	4.49x10-5	0.0012	*HLA-DRA, DYNC1I2, TUBB2A, ATP6V1A, HLA-E, HLA-F, TAP1, TUBB6, DYNC1I1, HLA-DPB1, HLA-A, HLA-DRB1, HLA-DPA1, PLA2R1, HLA-DQB1, ITGB3, ATP6V0A4, TUBAL3, TAP2, HLA-C, DYNC2H1, TUBB8, TUBA3D, COMP, ATP6V0D1, ATP6V0E2, HLA-DQA2, SEC61B, PIK3C3, HLA-DQA1, THBS2, VAMP3, FCGR3B, COLEC11, ITGB2, HLA-DMA, HLA-B, HLA-G, SCARB1, HLA-DOA, HLA-DOB, HLA-DRB5*
ECM-receptor interaction	28	13.34	2.10	5.80x10-5	0.0013	*LAMA4, SV2C, COL6A2, HSPG2, COL4A2, LAMC1, AGRN, THBS2, COL11A2, COL1A1, TNXB, RELN, LAMA2, COL6A3, GP5, COL6A1, COL2A1, COL5A1, LAMB1, ITGB3, COL4A1, ITGA11, GP6, COL5A3, ITGA1, ITGA9, CD44, COMP*
Other types of O-glycan biosynthesis	17	6.91	2.46	0.0002	0.0038	*UGT1A1, GXYLT2, UGT1A4, UGT2B15, UGT1A10, UGT1A7, MGAT5B, POMGNT1, UGT1A5, ST3GAL3, UGT1A3, UGT2B17, FUT9, UGT1A9, UGT1A6, UGT1A8, ST6GAL2*
Ascorbate and aldarate metabolism	12	4.02	2.99	0.0002	0.0038	*UGT1A1, UGT1A4, UGT1A9, UGT2B15, UGT2B17, UGT1A10, UGT1A6, UGT1A8, ALDH7A1, UGT1A7, UGT1A5, UGT1A3*
Steroid hormone biosynthesis	19	8.68	2.19	0.0005	0.0087	*UGT1A1, CYP19A1, UGT1A4, UGT2B15, CYP7B1, UGT1A10, UGT1A7, UGT1A5, HSD3B2, UGT1A3, CYP11B2, UGT1A9, UGT2B17, SRD5A3, UGT1A6, UGT1A8, HSD17B12, AKR1C4, CYP1B1*
Starch and sucrose metabolism	18	8.36	2.15	0.0008	0.0128	*UGT1A1, GBE1, GAA, UGT1A4, UGT2B15, UGT1A10, PYGL, UGT1A7, UGT1A5, UGT1A3, PYGB, GCK, UGT2B17, UGT1A9, HK2, UGT1A6, UGT1A8, AGL*

Asthma	11	4.34	2.54	0.0019	0.0282	*HLA-DRB1, HLA-DRA, HLA-DPA1, HLA-DMA, HLA-DQA2, HLA-DQA1, HLA-DQB1, HLA-DOA, HLA-DPB1, HLA-DOB, HLA-DRB5*
Staphylococcus aureus infection	17	8.52	2.00	0.0029	0.0402	*C4B, HLA-DRA, HLA-DQA2, HLA-DQA1, C4A, KRT10, FCGR3B, HLA-DPB1, HLA-DMA, HLA-DPA1, ITGB2 ,HLA-DRB1, C1QA, HLA-DQB1, HLA-DOA, KRT10, HLA-DQA1, HLA-DOB, HLA-DRB5*
Intestinal immune network for IgA production	15	7.23	2.07	0.0033	0.0429	*HLA-DRB1,HLA-DRA,HLA-DPA1,HLA-DMA,HLA-DQA2,HLA-DQA1,HLA-DQB1,CD86,TNFSF13B,CD80,HLA-DOA,TNFRSF17,HLA-DRB5,HLA-DPB1,HLA-DOB*
Mucin type O-Glycan biosynthesis	11	4.66	2.36	0.0037	0.0453	*GALNT3, GALNT1, GALNTL1, GALNT2, GALNT9, GALNT6, WBSCR17, GALNT10, GALNT7, ST3GAL1, GALNTL4*

P-values have been adjusted for multiple testing using Benjamini-Hochberg
